# Transcriptome Analysis Reveals Downregulation of Urocortin Expression in the Hypothalamo-Neurohypophysial System of Spontaneously Hypertensive Rats

**DOI:** 10.3389/fphys.2020.599507

**Published:** 2021-03-17

**Authors:** Andrew Martin, Andre S. Mecawi, Vagner R. Antunes, Song T. Yao, Jose Antunes-Rodrigues, Julian F. R. Paton, Alex Paterson, Michael Greenwood, Olivera Šarenac, Bojana Savić, Nina Japundžić-Žigon, David Murphy, Charles C. T. Hindmarch

**Affiliations:** ^1^Bristol Medical School: Translational Health Sciences, Dorothy Hodgkin Building, University of Bristol, Bristol, United Kingdom; ^2^Laboratory of Neuroendocrinology, Department of Biophysics, Paulista School of Medicine, Federal University of São Paulo, São Paulo, Brazil; ^3^Department of Physiology and Biophysics, Institute of Biomedical Sciences, University of São Paulo, São Paulo, Brazil; ^4^Florey Institute of Neuroscience and Mental Health, Parkville, VIC, Australia; ^5^Department of Physiology, School of Medicine of Ribeirão Preto, University of São Paulo, Ribeirão Preto, Brazil; ^6^Manaaki Mānawa, The Heart Research Centre, University of Auckland, Auckland, New Zealand; ^7^Faculty of Medicine, Institute of Pharmacology, Clinical Pharmacology and Toxicology, University of Belgrade, Belgrade, Serbia; ^8^Queen’s Cardiopulmonary Unit, Department of Medicine, Translational Institute of Medicine, Queen’s University, Kingston, ON, Canada

**Keywords:** hypothalamo-neurohypophyseal system, transcriptome, SHR, UCN, spectral analysis, microarray, hypertension

## Abstract

The chronically increased blood pressure characteristic of essential hypertension represents an insidious and cumulative risk for cardiovascular disease. Essential hypertension is a multifactorial condition, with no known specific aetiology but a strong genetic component. The Spontaneously Hypertensive rat (SHR) shares many characteristics of human essential hypertension, and as such is a commonly used experimental model. The mammalian hypothalamo-neurohypophyseal system (HNS) plays a pivotal role in the regulation of blood pressure, volume and osmolality. In order to better understand the possible role of the HNS in hypertension, we have used microarray analysis to reveal differential regulation of genes in the HNS of the SHR compared to a control normotensive strain, the Wistar Kyoto rat (WKY). These results were validated by quantitative reverse transcription-polymerase chain reaction (qRT-PCR). One of the genes identified and validated as being downregulated in SHR compared to WKY was that encoding the neuropeptide urocortin (Ucn). Immunohistochemical analyses revealed Ucn to be highly expressed within magnocellular neurons of the PVN and SON, with pronounced localisation in dendritic projections containing oxytocin and vasopressin. When Ucn was overexpressed in the PVN of the SHR by *in vivo* lentiviral mediated gene transfer, blood pressure was unaffected but there were significant, transient reductions in the VLF spectra of systolic blood pressure consistent with an action on autonomic balance. We suggest that Ucn may act, possibly via dendritic release, to subtly regulate neurohumoral aspects of arterial pressure control.

## Introduction

An estimated 17.3 million people die as a result of cardiovascular diseases every year, with 80% of these deaths occurring in low- and middle-income countries (World Heart Federation [WHF], 2017). A major contributor to these deaths is high blood pressure (hypertension), which triples the risk of coronary heart disease, increases the chance of stroke sevenfold, and of congestive heart failure by sixfold. According to World Health Organisation estimates, 1.13 billion people worldwide have hypertension, two-thirds of whom live in low- and middle-income countries (Hypertension Affects Everyone^1^; World Health Organization [WHO], 2019). Despite the prevalence of essential hypertension, little is known about its aetiology, although a number of risk factors are well recognised. Indeed, we routinely assert the truism that primary hypertension is the result of a mosaic of modifiable and un-modifiable genetic and lifestyle risk factors that conspire to change the blood pressure set point. Beyond that, we know very little about the fundamental mechanisms by which these risk factors interact to engender hypertension. That said, it is useful to consider the development of high blood pressure to be a continuum, with altered autonomic nervous activity preceding the phenotype (i.e., pre-hypertensive) and then increasing levels of BP indicating the severity of the disease. Within this context, we have focussed on the central neuronal changes evident in hypertension as these occur prior to the onset of hypertension and are indeed recognised prognostic indicators and primary drivers (Mann, 2018).

The genetically programmed spontaneously hypertensive rat (SHR) (Trippodo and Frohlich, 1991) is an established model that is widely used. The strain is fully inbred, and hypertension develops with maturity without the need for dietary or environmental stimuli (Trippodo and Frohlich, 1991). Much is known about the causes and consequences of hypertension in the SHR, particularly central nervous control. These have parallels with man, such as autonomic dysfunction (heightened sympathetic activity and increased BP variability), end organ damage (stroke, cardiac hypertrophy, renal, and endothelial dysfunction) (Trippodo and Frohlich, 1991) and sensitivity to angiotensin converting enzyme inhibitors (Morishita et al., 1995). Importantly, we have shown that sympathetic nerve activity in juvenile, pre-hypertensive SHR is raised prior to the overt onset of hypertension (Simms et al., 2009).

The hypothalamo-neurohypophysial system (HNS), consisting of the neurones of the supraoptic nucleus (SON) and the paraventricular nucleus (PVN), is an important integrative structure that regulates co-ordinated neurohumoral responses to homoeostatic perturbation that can potent effects on blood pressure and has been implicated in the aetiology of hypertension (Qin et al., 2018; Saxena et al., 2018; Zhou et al., 2019). Endocrine effects are mediated through the axonal projections from SON and PVN magnocellular neurones (MCNs) to the posterior pituitary (PP), which are crucially involved in the regulation of osmotic stability and blood volume and, hence arterial pressure (Burbach et al., 2001; Antunes-Rodrigues et al., 2004; Mecawi Ade et al., 2015). The antidiuretic hormone vasopressin (VP) and the natriuretic hormone oxytocin (OT) are important regulators of cardiovascular homoeostasis (Japundžić-Žigon et al., 2020), and both are synthesised as separate prepropeptide precursors in the MCN cell bodies of the SON and PVN (Brownstein et al., 1980; Burbach et al., 2001). These precursors are processed during anterograde axonal transportation via the median eminence to terminals in the PP, where biologically active VP and OT is stored until mobilised for secretion into the circulation by MCN electrical activities evoked by hyperosmolality (Bourque et al., 1994; Bourque, 1998). A rise in plasma osmolality is detected by intrinsic MCN osmoreceptor mechanisms (Bourque et al., 1994, 2002; Bourque, 1998; Zhang and Bourque, 2003) and by specialised osmosensitive neurones in the circumventricular organs (CVOs) that project to, and regulate, SON MCNs (Bourque et al., 1994; Bourque, 1998; McKinley et al., 2004). Upon release, VP travels through the blood stream to specific receptor targets located in the kidney where it increases the permeability of the collecting ducts to water, reducing the renal excretion of water, thus promoting water conservation and increasing blood volume (Breyer and Ando, 1994). VP also vasoconstricts blood vessels to increase peripheral resistance supporting circulation (Japundžić-Žigon, 2013). OT, as well as having well known reproductive roles, has natriuretic activity at the level of the kidney (Conrad et al., 1993). Dysfunction of the SHR endocrine HNS has been documented. It has been reported that SHRs drink more water than WKYs (Kraly et al., 1985), show hypertrophy of the PP (Pérez-Delgado et al., 2000) along with a higher VP content (DeVito et al., 1982; Sladek et al., 1988) and elevated plasma VP levels (Morris et al., 1981; van Tol et al., 1988). Evidence has been presented that suggests that these are primary abnormalities in the SHR, not just a response to the hypertension (Sladek et al., 1988) and the SON and PVN are therefore highly pertinent structures for interrogation in relation to the abnormalities of hypertension.

Whilst the SON is a homogenous collection of MCNs, the PVN is divided into a lateral and more medial sub-division of MCNs and smaller parvocellular neurons, respectively. Through descending projections from parvocellular neurones to the brainstem, notably the rostral ventrolateral medulla (RVLM), and intermediolateral cell column of the spinal cord (Sawchenko and Swanson, 1982; Hardy, 2001), the PVN regulates changes in sympathetic nerve activity involved in the regulation of both arterial pressure and blood volume (Yang and Coote, 1999; Badoer, 2001; Hardy, 2001; Coote, 2005). Interestingly, the PVN excitatory drive to the RVLM appears to be heightened in the SHR compared to the normotensive WKY (Allen, 2002). Conversely and consistent with activation of PVN pre-motor activity, GABA drive in PVN is decreased in hypertensive models (Martin and Haywood, 1998).

In the context of the importance of the HNS in the integrative control of arterial pressure, we have used microarrays to profile the transcriptomes of the SON and the PVN from adult SHR and the WKY rats. Comparing these datasets, we have identified genes that are expressed at significantly different levels in these two rat strains which we suggest might be potential players involved in the neuroendocrine or pre-motor components of the hypertensive state. One of these genes, that encoding the 40 amino acid peptide Urocortin (Ucn), is of particular interest as the literature implies roles in metabolic and cardiovascular function (Skelton et al., 2000; Latchman, 2002; Takahashi et al., 2004; Stengel and Taché, 2014). We thus validated a reduction of Ucn expression in SHR compared to WKY, and we have investigated the impact of its overexpression in the PVN of SHR rats on cardiovascular balance.

## Materials and Methods

### Animals

Experimental procedures were approved by the University of Bristol Ethical Review Committee and performed under the authority of a Project Licence issued by the United Kingdom Home Office in accord with the Animals (Scientific Procedures) Act 1986. Adult (224–311 g, 11–14 weeks old) or juvenile (84–109 g, 4–6 weeks old) WKY/NHsd and SHR/NHsd rats were obtained from Harlan Sera-lab (now Envigo). Animals were maintained in standardised conditions (temperature 22 ± 1°C, humidity 50 ± 5% v/v, diurnal cycle of 10 h light and 14 h dark; lights on at 07:00), housed in groups of no less than two animals per cage. Animals were given access to food (standard laboratory rat chow) and water *ad libitum*. All animals were killed between 9:00 and 13:00 h.

All experimental procedures carried out in Belgrade conformed to the European Communities Council Directive 1986. All experimental protocols were approved by the Faculty of Medicine University of Belgrade Ethics Review Board. Experiments were performed on 15–17-week-old male SHRs (University of Belgrade animal facility). All animals used ranged in weight between 252–287 g at time of use and were housed individually, and under standard conditions (12 h/12 h light-dark cycle, 21 ± 2°C, humidity 60 ± 5%). Standard pelleted rat chow and water was available *ad libitum* during the entire experimental time-course.

The number of rats in each protocol was calculated statistically taking into account intra-group variability, using the “Power Sample Size Calculation”^2^ for power of 90% and type I error probability of 0.05.

### Tail Cuff Blood Pressure Measurement

Five days prior to collection and processing of tissue for microarray analysis, systolic blood pressure measurements were obtained. Animals were subject to prior conditioning before measurements were taken. Rats were pre-warmed under a heating lamp for 5 min prior to being placed in a restraining tube. An inflatable tail cuff was placed at the base of the tail and blood pressure was measured using an Advanced NIBP Blood Pressure Monitor (Harvard Apparatus).

### Hormone Analysis

Plasma was obtained from trunk blood samples collected from trunk blood in chilled, peptidase inhibitor coated vacutainers by centrifugation at 3,000 rpm, 4°C for 20 min. Plasma angiotensin II (Ang II) and atrial natriuretic peptide (ANP) concentration was quantified using radioimmunoassay (RIA) procedure as previously described (Mecawi et al., 2013). Data was expressed as pg/ml and the sensitivities of RIA and intra- as well as interassay coefficients of variation for were 0.7 pg/ml, 4.8 and 10% for ANP and 0.39 pg/ml, 8.7 and 11.2% for Ang II.

### Affymetrix GeneChip^®^ Analysis

Transcriptome services were provided by Source−Bioscience. Methods have been described (Hindmarch et al., 2006). Age-matched adult (11–13 weeks old) male inbred WKY rats and SHRs were stunned and decapitated with a small animal guillotine (Harvard Apparatus). Brains were rapidly removed and placed in an ice-cold brain matrix (ASI Instruments). Two 1 mm of thickness section of the hypothalamus were obtained using the optic chiasm as a landmark and the SON and PVN was carefully dissected from these with the aid of a dissecting microscope (Leica). RNA was extracted using QIAzol Lysis Reagent (Qiagen) and the aqueous phase was removed after centrifugation through a Phase Lock Gel column (Eppendorf). Total RNA was purified with RNeasy Micro Kit MinElute Spin Columns (Qiagen). Samples that met quality control criteria were used as templates for cRNA synthesis and Biotin labelling, incorporating a single round of linear amplification using the Ambion Message Amp II aRNA kit (Ambion). Following fragmentation, sample were hybridised to Rat Genome 230 plus 2.0 GeneChip^®^ arrays for 16 h in the Affymetrix GeneChip^®^ Hybridisation Oven 6400 (Affymetrix). Following hybridisation, the GeneChip^®^ arrays were stained and washed on the GeneChip^®^ Fluidics Station 400 (Affymetrix), and fluorescent signals were detected using the Affymetrix GeneChip^®^ Scanner 3000 (Affymetrix). These data were initially documented using Affymetrix Microarray Suite software (MAS 5.0), which generates an expression report file that lists the quality control parameters. All of these parameters were scrutinised to ensure that array data had reached the necessary quality standards (Hindmarch et al., 2006). Data were uploaded into GeneSpring software version 7.0 (Agilent) for normalisation and high-level analysis (Hindmarch et al., 2006). Data were filtered so that only probe sets that are considered to be present in all the chips from at least one of the experimental groups (SON-SHR or SON-WKY, PVN-SHR or PVN-WKY) were available for statistical analysis. A Welch *t*-test was performed which assumed unequal variance and included the Benjamini and Hochberg multiple testing correction, limiting those genes identified by chance to just 5%. Finally, a twofold difference cut-off was applied to the data. See section “Results” for details. All raw data have been submitted to the NBCI Gene Expression Omnibus (GEO)^3^.

### Quantitative Reverse Transcription PCR (qRT-PCR)

Adult and juvenile WKY and SHR rats were killed by stunning followed by decapitation using a small animal guillotine (Harvard Apparatus). Brains were removed and were snap-frozen using powdered dry ice and stored at −80C for later processing. Specific hypothalamic nuclei were punched from sequential caudal-rostral brain sections (60 μm) obtained using a Leica Microsystems CM1900 cryostat (Leica). Nuclei were located using toluidine blue (Sigma Aldrich; 0.1% w/v in 70% v/v ethanol) staining, in conjunction with a microscope and brain map (Paxinos and Watson, 2013). Microtissue punches were obtained using a 1 mm diameter micro punch (Fine Science Tools Inc). RNA extraction and cDNA synthesis protocols have been described (Greenwood et al., 2014). Primer pairs were sourced from Eurofins MWG Operon. Primer sets are detailed in Supplementary Table 1. All primer sets were tested to achieve a high degree of efficiency (>95%). Biological samples were analysed for target gene expression in duplicate or triplicate technical replicates. Analysis of relative gene expression (fold-change) between biological samples followed the Pfaffl Method (Pfaffl, 2001) using the Rpl19 gene as a normalisation standard. Fold-change values were transferred into GraphPad Prism Software (Version 6.0) where statistical analysis tests were applied using 2-Way ANOVA (Analysis of Variance) with Tukey *Post Hoc* correction applied.

### Immunohistochemistry

Animals were deeply anesthetised using intraperitoneal pentobarbital sodium (Euthatal; 100 μg/kg) as demonstrated by a complete absence of withdrawal responses to noxious pinching of the tail or a paw, and heavily depressed breathing. Animals underwent transcardial perfusion at a flow rate of approximately 20 mL/min with 250 mL ice-cold PBS followed by 400 mL ice-cold 4% (w/v) PFA to fix tissue. Whole brains and pituitary glands were removed and placed into ice-cold 4% (w/v) paraformaldehyde (PFA), then stored for 12 h at 4°C after which the PFA was replaced with 30% (w/v) sucrose solution until completely infused. Tissue samples were then frozen over dry ice and stored at −80C for future use. Caudal-rostral tissue sections (20–40 μm thick) were obtained using a Leica Microsystems CM1900 cryostat (Leica Microsystems). Sections were placed free-floating in 24-well tissue culture plates containing ice-cold phosphate buffered saline (PBS) prior to immunohistochemical processing. Pituitary sections were placed onto Poly-L-Lysine coated microscope slides (PolySciences Inc.) and were circled using a PAP hydrophobic barrier pen (Sigma Aldrich). Immunohistochemical and immunofluorescence methods have been described (Greenwood et al., 2014). Details of primary and secondary antibodies are found in Supplementary Table 3. Mounted sections were imaged using a Leica SP5-AOBS confocal laser-scanning microscope in conjunction with Leica Microsystems LAS AF software. Images were acquired via a sequential scanning method to avoid cross immunofluorescence. Processing of images acquired through confocal microscopy was facilitated using ImageJ/Fiji imaging software.

### Quantitative Image Analysis

For each structure of interest (SON, PVN, pituitary), three slices (20 μm thick) from each were imaged. A region of interest (87.50 × 387.50 microns) was selected for analysis. Through the use of the “Sum Slices” function in ImageJ software, the series of z-stack images were combined into a single 32-bit grey-scale image that represents the sum of intensity values of all pixels at a given *x, y* position through the z-stack. A lower-limit threshold value was set for each image to be analysed in order to exclude background. The same threshold level was standardised across all samples of the nuclei for comparison; SON = 400, PVN = 128, pituitary = 10 (grey-scale units). All pixels with values below threshold were designated “not a number” (NaN, indicating no numerical intensity value) thereby excluding them from the analysis. For histogram analysis, a standardised bin width of 1 was chosen, allowing for the number of pixels (Count) per bin (Intensity) with a sum grey-scale unit of intensity >400 (Intensity) to be counted. Count and Intensity values were then exported from Fiji into Excel for normalisation and statistical analysis. To normalise for potential differences in total area measured between samples (after threshold application), the total area value (pixels) for each image were divided by the image mean intensity value (grey-scale units). These Total Area/Mean values for each sample were then averaged (Mean) across the group (*n* = 3) and a statistical comparison (Student’s *t-*test, 1 tailed, type 1) was performed between WKY vs. SHR.

### Colocalisation Analysis

Fiji software (running the Coloc2 plugin) was utilised to quantitatively assess colocalisation of Ucn with either VP/OT, through a Pearson Correlation Coefficient (***r****_*p*_*) alongside Manders overlap coefficient (MOC) tests. Prior to performing Pearson and Manders tests, background was corrected using a rolling ball pixel radius (standardised between compared images) and region(s) of interest (ROI) were carefully identified (depending on the objective of the comparison). Colocalisation is reported here through; *r*_*p*_ values (without threshold) alongside Manders tM1 and tM2 significance test outputs.

### Lentiviral Vectors

An RT-PCR-derived and Sanger sequence checked full length Ucn cDNA sequence was cloned into the pRRLsinpptCMV.GFPpre lentiviral vector (Addgene) by replacement of the endogenous eGFP sequences to generate pRRLsinpptCMV.Ucnpre. Overexpression of Ucn (experimental group; LV-Ucn) or eGFP (control group; LV-eGFP) is facilitated in these vectors by the cytomegalovirus (CMV) promoter/enhancer. Viruses were generated by transient transfection of the transfer vector together with three separate packaging plasmids (pMDLg/pRRE, pRSV-Rev, PMD2.G; Addgene) into HEK293T cells by the calcium phosphate method, as previously described (Panyasrivanit et al., 2011). Media was collected at 48 and 72 h after transfection, cell debris was removed by centrifugation, and the supernatant was filtered through a 0.45 μm filter. High-titer lentiviruses were produced by centrifugation at 6,000×g for 16 h (400 ml), followed by ultracentrifugation of the resuspended pellet (10 ml of PBS) for 1.5 h at 50,000×*g*. The viral pellet was resuspended in 150 μL of prewarmed PBS and stored in 5 μL aliquots at −80°C. Viral titres were determined by counting Ucn- or GFP-positive or cells at day 3 following infection of HEK293T cells. Calculated titres were 7.31 × 10^7^ and 9.54 × 10^7^ TU/mL for LV-eGFP and LV-Ucn, respectively. Overexpression of Ucn protein was confirmed in HEK293T cells transduced with the LV-Ucn lentivirus by both immunocytochemistry and Western blotting (not shown).

### Measurement of Cardiovascular Parameters in SHR Rats Over-Expressing Ucn in the PVN

Rats underwent two surgical procedures in total, with a 7 days post-surgical-period following each procedure to allow for full recovery to health before any blood pressure data was recorded. General anaesthesia for both procedures was administered using a combined mixture of ketamine (100 mg/kg, im) and xylazine (10 mg/kg, im). In order to prevent infection, neomycin and bacitracin were sprayed topically, and the rats received gentamicin parenterally (25 mg/kg i.m.) 3 days before, and again on the day of surgery. Carprofen (Rimadyl; 5 mg/kg/day, s.c.) as an analgesic was administered on the day of surgery and for the next 2 days.

The first surgery involved the implantation of a radiotelemetry device (TA11-PA C40; DSI). A 3 cm-long medial abdominal incision was made, and the intestine was retracted to expose the abdominal aorta. The tip of the catheter of the radiotelemetric probe was inserted into the abdominal aorta using a 21G needle. The inserted catheter was fixed with 3 M Vetbond^TM^ and tissue cellulose patch (DSI). The transmitter was attached to the anterior abdominal wall and the wound was closed by suture Following a minimum of 7 days recovery post-telemeter implantation, a baseline ambulatory recording of 30 min was performed for each animal using the telemetric recording system (detailed below). This acted as an experimental quality-control step to determine if the animal was hypertensive, with only those showing a SBP of >140 mmHg progressing to viral transfection.

For injection of lentiviruses into the PVN, the head of the anaesthetised rat was mounted in the stereotaxic frame (David Kopf Instruments) and a 2 cm rostral-caudal midline incision was made to expose the skull. A dental drill (Quayle Dental) was used to drill 1 mm holes through the skull to expose the underlying dura mater and brain. Based on the stereotaxic coordinates of the PVN (1.8 mm caudal from bregma, 0.4 mm lateral from midline, 7.4 mm beneath the skull) (Paxinos and Watson, 2013), a glass micropipette was used to infuse 1 μL of lentiviral suspension (either titre-matched LV-eGFP or LV-Ucn) at a rate of 0.1 μL/min. Rats were left to recover for 7 days.

Following recovery, arterial BP was recorded using a DSI recording system at time-point intervals of 7, 10, 14, 19, and 21 days-post transfection. At time of recording, arterial BP was digitised at 1,000 Hz using Dataquest A.R.T. 4.0 software (DSI).

At the end of the experiment, animals were sacrificed by decapitation using a small animal guillotine (Harvard Apparatus). Brains and pituitary were removed and stored at −80C for subsequent analyses. Successful transfection of lentivirus into the PVN by microinjection was retrospectively validated through the use of RT-qPCR. Only data from such validate animals was used.

### Processing and Analysis of Blood Pressure Signals

Values for SBP, DBP, pulse interval (PI) and heart rate (HR = 60/PI) were calculated as maximum, minimum and dP/dt_*max*_ inter-distance of the arterial pulse pressure wave, respectively. Prior to spectral analysis, signals were re-sampled at 20 Hz and subjected to 9-point Hanning window filter and linear trend removal (Milutinovic et al., 2006; Stojicic et al., 2008). BP spectra were obtained through use of specially customised software utilising a fast Fourier transformation algorithm on 30 overlapping 2048 point time series involving 410 s (∼7 min) registration period of SBP, DBP, and HR. Power spectra of SBP/DBP (mmHg^2^) and HR (bpm) for FFT segments were calculated for the whole spectrum [Total (SBP/DBP/HR); 0.0195–3 Hz] and over the following three frequency ranges: VLF; 0.0195–0.195 Hz, LF; 0.195–0.8 Hz, and HF; 0.8–3 Hz. Cardiovascular parameters are expressed as mean ± S.E.M. Statistical tests were performed in Graph Pad Prism 6 software (GraphPad Software Inc.). Analysis used one-way ANOVA with repeated measures followed by Bonferroni *post hoc* test for comparisons of variables in time and between groups (one independent factor/variable: UCN expression on BP in SHR). All BP and HR parameters were assessed using the same test.

Evaluation of baroreceptor reflex sensitivity (BRS) was assessed through spontaneous baroreceptor reflex sequences analysis, these comprised a stream of consecutively increasing/decreasing SBP samples, followed by a stream of increasing/decreasing PI samples, were delayed by 3, 4, or 5 beats in respect to SBP. A sequence length threshold was set to 4 beats (Turuk Turukalo et al., 2011). BRS (ms/mmHg) was analysed via linear regression coefficient averaged over all sequences (PI = BRS × SBP + constant; where fitting of the curve is performed in a “least-squares” sense). Sequences were taken from 7 min registration periods. Sequence coverage area (SCA) (operating range) was also calculated, this representing a set region of the SBP PI plane, in-between lowest and highest sequence data values across both dimensions (5% outlier points were omitted) (Bajić et al., 2010).

### Ucn ELISA

A sandwich enzyme-linked immunosorbant assay (ELISA) kit (Cloud-Clone Corp) was used to quantify Ucn in the sonicated pituitary homogenates (Soniprep 150 m, MSE) in accordance with the manufacturer’s instructions. The sensitivity range of this kit ranged from 5.5 to 1,000 pg/mL. The ELISA plate was analysed using a BioRad impark Plate Reader (BioRad, United Kingdom) reading at 450 nm. Optical density (O.D.) data was analysed using Microsoft Excel spreadsheet, through; average (mean) of triplicate wells subtracted from standard BLANK data (background). A log-log standard curve of concentration vs. absorbance was constructed by plotting mean O.D. vs. concentration of standards used. From this an average (mean) concentration of Ucn was calculated for each group. A Student’s *t*-test was applied to discern statistical differences between groups (*p* ≤ 0.05).

## Results

### Physiological and Endocrine Measures

**FIGURE 1:**
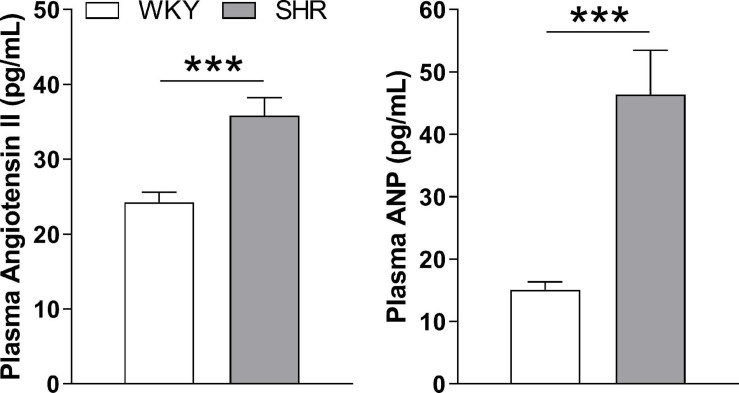
Circulating levels of angiotensin II and atrial natriuretic hormone (ANP) I plasma from WKY and SHR rats. ***p ≤ 0.001 (*n* = 10).

As measured by tail-cuff, adult SHR rats exhibited a higher systolic blood pressure than WKY rats (200 ± 4 vs. 156 ± 3 mmHg; *p* < 0.05, *n* = 25). Consistent with their hypertension, they also had higher plasma angiotensin II and atrial natriuretic peptide levels (Figure 1).

### Microarray Analysis of the Transcriptomes of the WKY and SHR SON and PVN

We interrogated Affymetrix GeneChip^®^ Rat Genome 230 2.0 Arrays with targets derived from PVN and SON, of WKY and SHR rats (*n* = 5, with each group comprising 5 pooled PVNs or SONs). Normalised data were subjected to high level analysis using GeneSpring^®^ software version 7.0 (Agilent Technologies). We first compiled lists of genes called present (P) in 5 out of 5 replicates in each tissue sample from each strain. All marginal or absent calls were excluded. These gene lists are transcriptome catalogues that, with a high degree of confidence, represent comprehensive descriptions of the RNA populations expressed in the WKY PVN (Supplementary Table 2A), SHR PVN (Supplementary Table 2B), WKY SON (Supplementary Table 2C) and SHR SON (Supplementary Table 2D). We then used GeneSpring^®^ to combine appropriate P gene lists to produce experimental gene lists for two-way comparative statistical analysis of each brain region (WKY vs. SHR) using previously normalised data. Note that some of these genes, although, by definition, called present in all of the samples of one experimental condition, may well be called absent or marginal in some or all of the samples of the other condition. These combined lists were then used as the basis for further filtering and statistical analysis. Firstly, for each comparison, lists were filtered to identify genes that are increased or decreased at least twofold. These lists were then separately used to statistically assess differences (Welsh *t*-test, *p* < 0.05, with Benjamini-Hochberg multiple test correction; the predicted false discovery rate of this protocol is ∼5% of identified genes). This analysis revealed 99 differentially regulated genes in the PVN (Supplementary Table 2E), of which 46 were more abundant in SHR compared to WKY, whilst 53 were more abundant in WKY compared to SHR. In the SON, there were 126 robustly differentially regulated genes (Supplementary Table 2F), of which 70 were more abundant in SHR compared to WKY, whilst 56 are more abundant in WKY compared to SHR.

### Gene Ontology Analysis of Transcriptome Data

The STRING database (string-db.org) was used to ask if the differentially expressed transcripts identified in the PVN of the SON could be classified according to enriched biological process gene ontology (GO) terms. Only one enriched term was significantly associated with the PVN data (GO:0065008, regulation of biological quality, FDR = 0.0279). In contrast, the SON data was associated with many biological process gene ontology (GO) terms (Supplementary Table 3), the most significant being regulation of biological quality (GO:0065008, FDR = 1.27e–05), response to stress (GO:0006950, FDR = 0.091) and regulation of blood pressure (GO:0008217. FDR = 0.0117). The 6 genes identified as being involved in blood pressure regulation are angiotensin II receptor associated protein (Agtrap), glutamyl aminopeptidase (Enpep), epoxide hydrolase 2 (Ephx2), natriuretic peptide receptor 3 (Npr3), phosphoinositide-3-kinase regulatory subunit 1 (Pik3r1) and Ucn.

### Validation of Differential Gene Expression in Juvenile and Adult SHR and WKY

**FIGURE 2:**
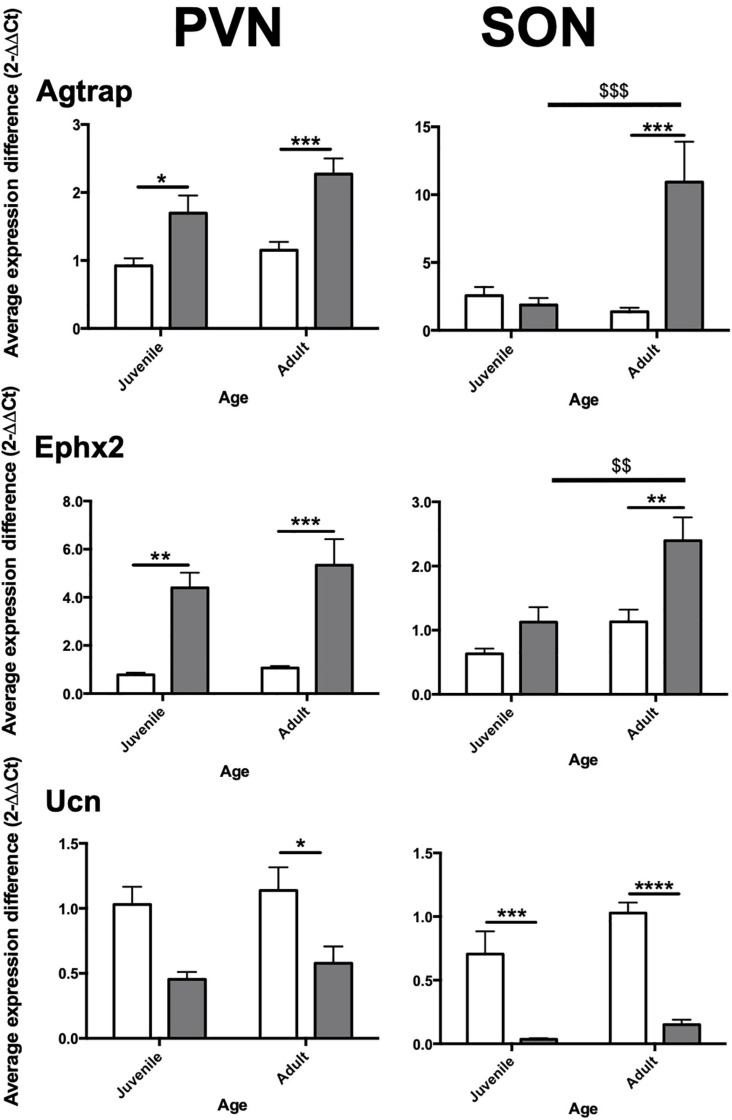
Relative expression of selected genes in PVN and SON in aged-matched WKY vs. SHR. Agtrap, Type-I Angiotensin II receptor-associated protein; Ucn, Urocortin; Ephx2, Soluble Epoxide Hydrolase gene. Juvenile = 6 weeks, adult = 12 weeks. Asterisks (*) indicate inter-strain significant differences within age. Dollar signs ($) indicate intra-strain significant differences between age. White bars: WKY. Black bars: SHR. Significance as follows; *p ≤ 0.05, **/$$*p* ≤ 0.01, ***/$$$ ≤ *p* ≤ 0.001, ****p < 0.0001. Error bars = Standard Error of Mean (S.E.M.).

Three blood pressure related genes were selected for RT-qPCR validation: Ephx2, Agtrap and Ucn. Validation was carried out in both juvenile and adult WKY and juvenile (pre-hypertensive) and adult SHR. Data are presented graphically in Figure 2.

In the PVN, we found Agtrap to be significantly increased in expression in juvenile SHR (+0.77-fold, *p* = 0.0454) and adult SHR (+1.12-fold, *p* = 0.0004) compared to age-matched normotensive counterparts. In the SON Agtrap expression was found to be markedly increased in adult SHR compared to adult WKY (+9.56-fold, *p* = 0.0002) but no similar change was seen in juvenile SHR (−0.68-fold, *p* = 0.99). In SHR, but not WKY, expression of Agtrap increased with age.

RT-qPCR analysis of gene of Ephx2 in PVN of both juvenile and adult SHR was robustly increased (+3.62, *p* = 0.004; +4.27, *p* = 0.0009, respectively) compared with that found in age matched WKY. Expression of Ephx2 in the adult SHR SON was significantly increased compared to adult WKY (+1.27-fold, *p* = 0.002). No significant difference was seen in juvenile animals (+0.50-fold, *p* = 0.382). In SHR, but not WKY, expression of Ephx2 increased with age.

In the PVN, no significant difference in Ucn expression was observed in juvenile SHR (−0.59-fold, *p* = 0.163) compared to juvenile WKY, however a significant decrease in expression was found in adult SHR (−0.61-fold, *p* = 0.042) compared to adult WKY. Analysis in the SON showed Ucn to be robustly decreased in both juvenile (−0.67-fold, *p* = 0.0002) and adult (−0.88-fold, *p* < 0.0001) SHR compared to WKY.

### Distribution of Ucn Peptide in the Adult WKY HNS

**FIGURE 3:**
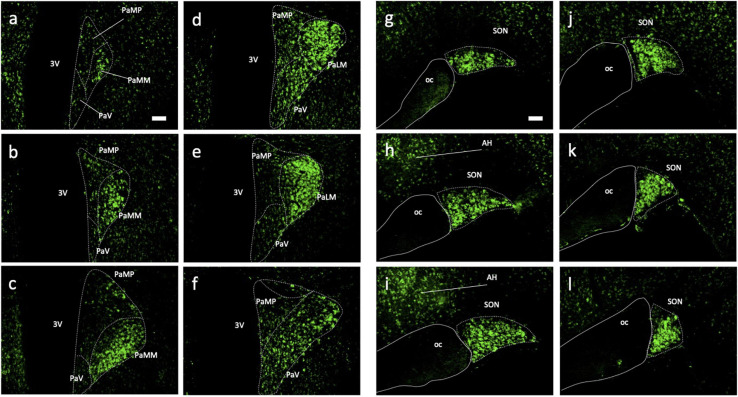
Immunohistochemical analysis of Urocortin expression in hypothalamic paraventricular and supraoptic nuclei of rat. Coronal sections (rostral-caudal) through rat hypothalamus showed high Ucn (green) expression within magnocellular portions of the PVN **(a–f)** and SON **(g–l)**. Lower expression was detected adjacent to both nuclei appearing localised to cell bodies only. 3V, Third Ventricle; PaMP, medial parvocellular PVN; PAMM, medial magnocellular PVN; PaV, ventral PVN; PaLM, lateral magnocellular PVN; oc, optic chiasm; PVN, paraventricular nucleus; SON, supraoptic nucleus; AH, anterior hypothalamic area; Ucn, Urocortin. Scale Bar = 100 μm.

**FIGURE 4:**
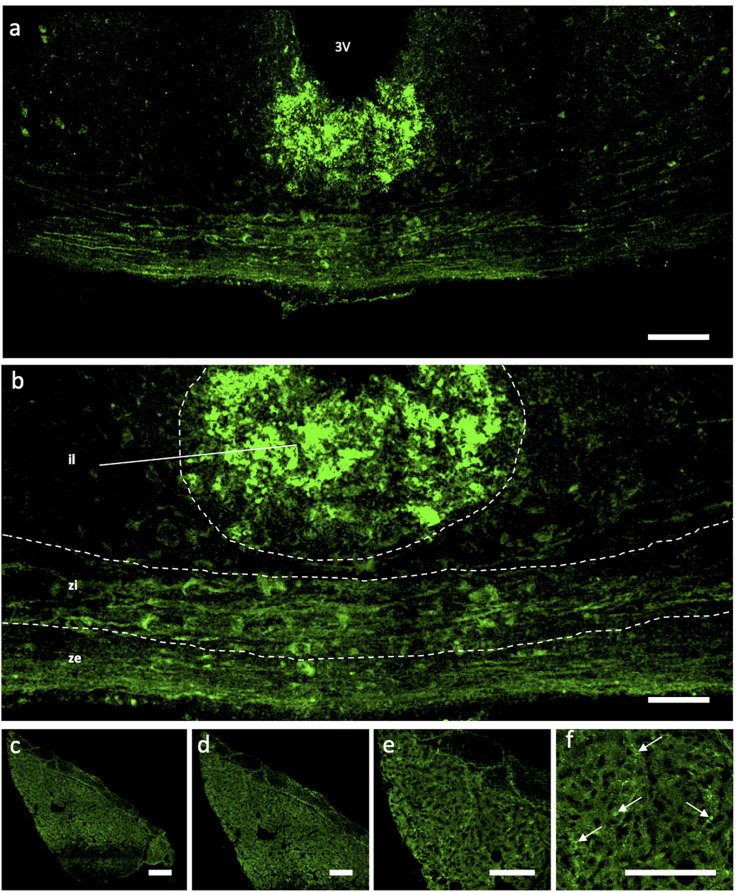
Immunohistochemical Analysis of Urocortin expression in Median Eminence and pituitary of rat. Expression of Ucn (green) was found within all areas (il, zi, ze) of the ME **(a,b)** as well as punctate staining located within the upper portion of the ME at the bottom of the 3V. Within the posterior lobe of the pituitary **(c–f)**, robust but diffuse Ucn expression was found throughout, with areas of particular punctate fluorescence (f; arrows). 3V, Third Ventricle; ME, median eminence; il, internal lamina; zi, zona interna; ze, zona externa; Ucn, Urocortin. Scale Bar = 100 μm.

Immunohistochemical analyses of Ucn peptide in the PVN (Figures 3a–f) revealed robust expression within the magnocellular divisions but only low level staining in parvocellular compartments. Strong expression was also seen in the SON (Figures 3g–l). In the median eminence (Figures 4a,b), diffuse as well as putative cellular Ucn expression was found in both zona interna (zi) and zona externa (ze). Furthermore, strong punctate Ucn expression was seen at the bottom of the 3rd ventricle within the superior aspect of the ME (internal lamina). In the posterior pituitary, expression was located seen throughout, appearing punctate in nature and often clustered (Figures 4c–f).

**FIGURE 5:**
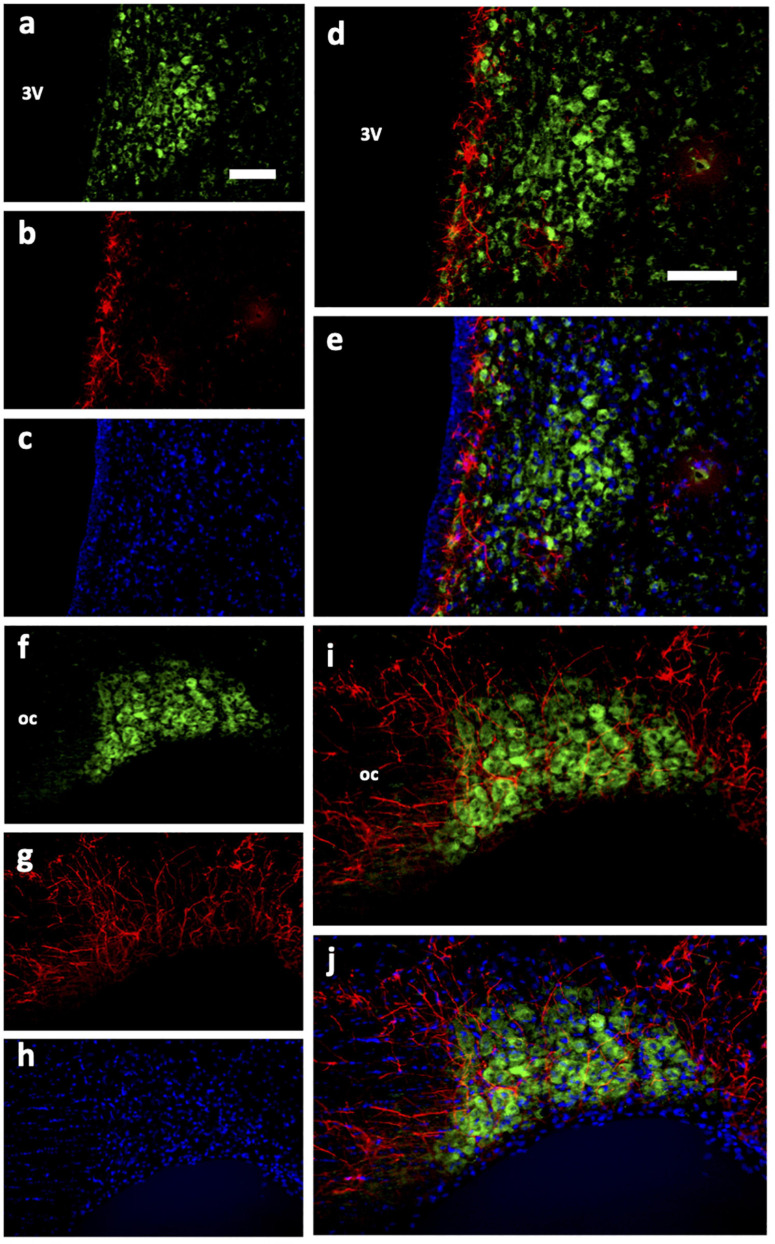
Immunohistochemical analysis of Urocortin expression with glial cells in PVN and SON. **(a,f)** Ucn. **(b,g)** GFAP. **(c,h)** DAPI. **(d,i)** Ucn + GFAP. **(e,j)** Ucn + GFAP + DAPI. Ucn, Urocortin; GFAP, glial fibrillary acidic protein; DAPI, 4′,6-diamidino-2-phenylindole. 3V, Third Ventricle; oc, optic chiasm. Scale Bar = 100 μm.

**FIGURE 6:**
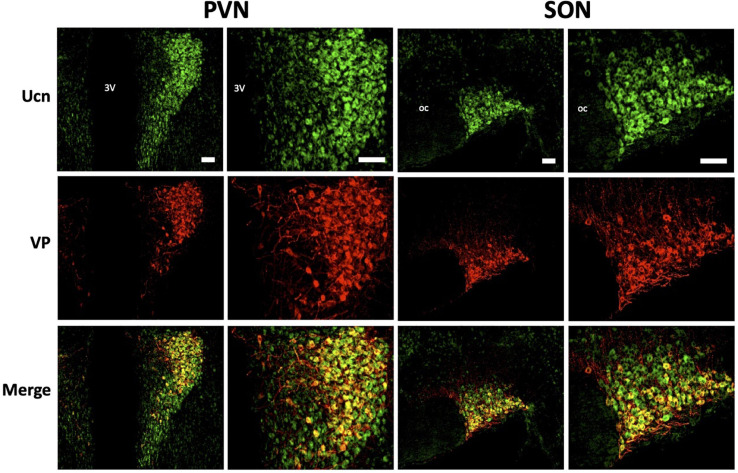
Representative images of colocalisation of Ucn with VP in SON and PVN. Double-labelling with antibodies directed against Ucn and VP showed an apparent level of colocalisation within a proportion of vasopressinergic magnocellular cells within the PVN and SON. Images of PVN show robust staining of Ucn within a large number of neurons in the lateral as well as ventral magnocellular divisions. Images of SON show robust staining of Ucn in both dorsal and ventral divisions, however, VP staining as well as colocalisation of Ucn and VP is more limited toward the ventral aspect of the nuclei. 3V, Third Ventricle; oc, optic chiasm; Ucn, Urocortin; VP, arginine vasopressin. Scale Bar = 100 μm.

**FIGURE 7:**
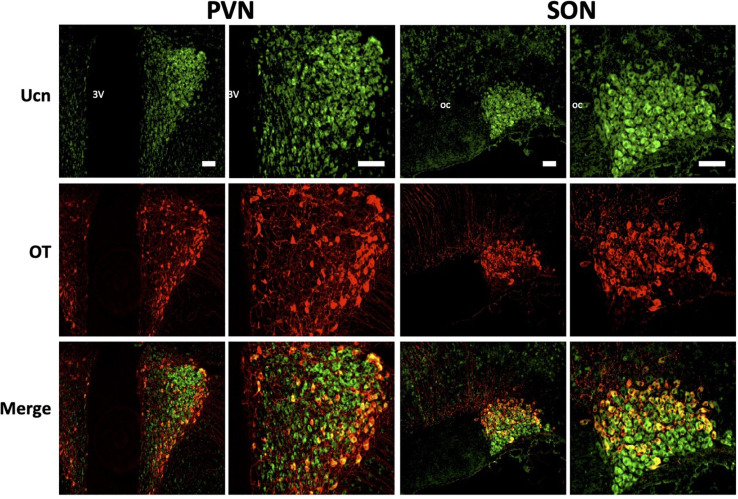
Representative images of colocalisation of Ucn with OT in SON and PVN. Double-labelling with antibodies directed against Ucn and OT showed an apparent level of colocalisation within a proportion of oxytocinergic magnocellular cells within the PVN and SON. Images of PVN show robust staining of Ucn within a larger number of neurons in the lateral as well as ventral magnocellular divisions, with number of OT stained neurons appearing lower and more diffuse within these divisions. Images of SON show robust staining of Ucn in both dorsal and ventral divisions, however, OTstaining, as well as colocalisation of Ucn and OT is more limited toward the dorsal aspect of the nuclei. 3V, Third Ventricle; oc, optic chiasm; Ucn, Urocortin; OT, oxytocin. Scale Bar = 100 μm.

**FIGURE 8:**
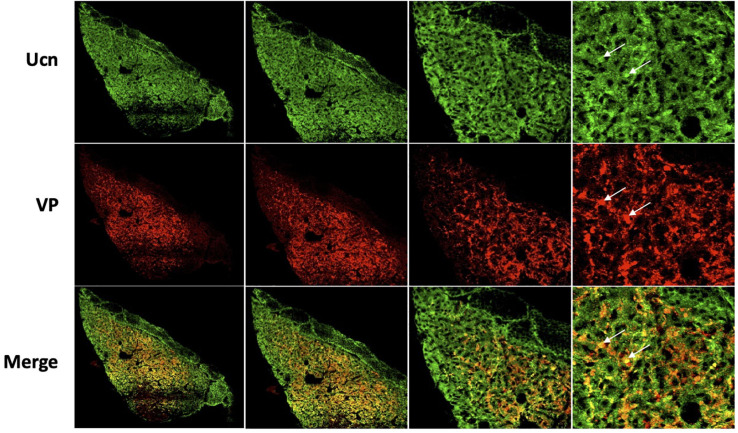
Representative images of colocalisation of Ucn with VP in the posterior pituitary. Double-labelling with antibodies directed against Ucn and VP showed an apparent level of colocalisation within a proportion of vasopressin containing terminals within the posterior pituitary. Images show staining of Ucn throughout the posterior pituitary with expression located within small punctate vasopressinergic regions. Ucn, Urocortin; VP, arginine vasopressin. Scale Bar = 100 μm.

We then determined the cell type and neurochemical phenotype of the Ucn expressing cells by double-labelling fluorescent immunohistochemistry using a variety of antigen markers. Ucn did not colocalise with GFAP, an astrocytic glial marker within either the PVN (Figures 5a–e) or SON (Figures 5f–j). In contrast, Ucn was prominently colocalised with both VP (Figure 6) and OT (Figure 7) in magnocellular neurones of both SON and PVN, but less so in the posterior pituitary (Figure 8).

Colocalisation analysis of Ucn and VP in the HNS found the strongest association to be within cells of the SON (***r****_*p*_* = 0.71), with a robust correlatory tM1 (manders tM1 threshold) and tM2 (tM1 = 0.859, tM2 = 0.881). In the PVN, colocalisation was also found in the lateral magnocellular division (*r*_*p*_ = 0.67) with reasonable support from Manders statistical test (tM1 = 0.693, tM2 = 0.815). Colocalisation between Ucn and VP was low in the posterior pituitary (*r*_*p*_ = 0.43, tM1 = 0.555, tM2 = 0.631). The degree of colocalisation of Ucn and OT was found to be highest in the magnocellular SON (*r*_*p*_ = 0.59), with a strong correlation between tM1 and tM2 (tM1 = 0.736, tM2 = 0.727). Less colocalisation was found in the lateral magnocellular division of the PVN (*r*_*p*_ = 0.49; tM1 = 0.855, tM2 = 0.841). Colocalisation between Ucn and OT was lowest in the posterior pituitary with punctate staining evident but at a lower level than observed for VP (*r*_*p*_ = 0.36, tM1 = 0.488, tM2 = 0.511).

**FIGURE 9:**
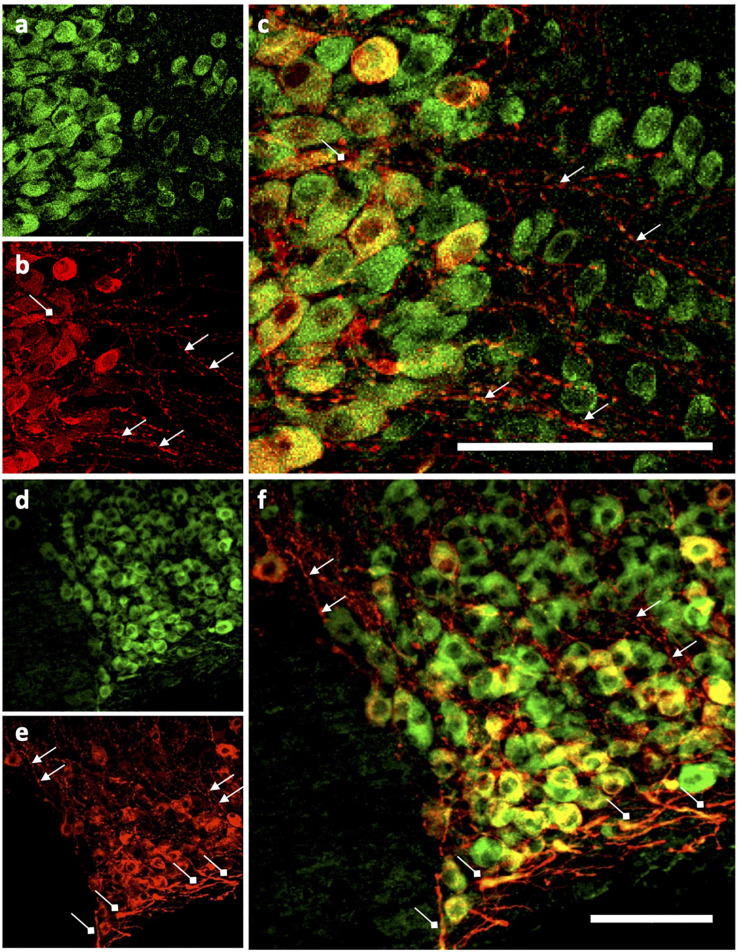
Ucn expression in vasopressinergic cells of PVN and SON. Ucn expression was noted in both vasopressinergic magnocellular PVN **(a–c)** and SON **(d–f)** cells. Ucn expression (green) can be clearly seen within what appear to be cell bodies in both nuclei. Vasopressinergic axonal projections (selection marked with triangle-tipped arrows) showed little expression of Ucn in either PVN or SON. Ucn expression did appear to localise within dendrites (selection marked with diamond-tipped arrows) of vasopressinergic neurons however as well as vasopressinergic cell bodies in both PVN and SON. SON **(a–c)** as follows; **(a)** Ucn, **(b)** VP, **(c)** Ucn + VP. PVN **(d–f)** as follows; **(d)** Ucn, **(e)** VP, **(f)** Ucn + VP. Ucn, urocortin; VP, arginine vasopressin. Scale Bar = 100 μm.

**FIGURE 10:**
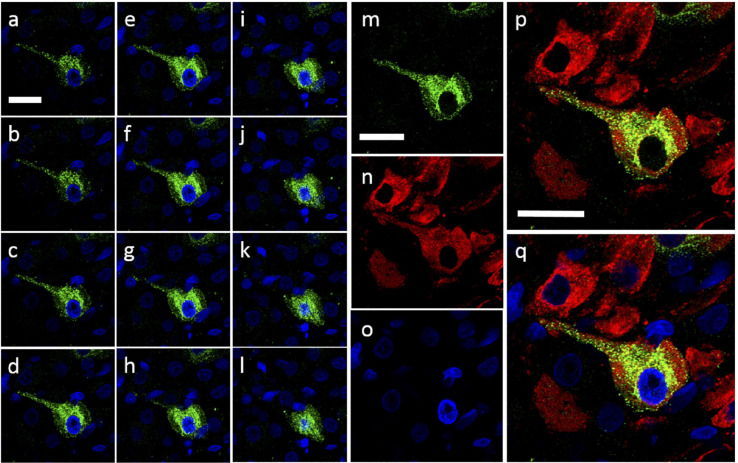
Intracellular Urocortin expression within cells of the caudal SON. **(a–l)** Coronal z-stack sequence (progressing caudal-rostral) through cell within the caudal rat hypothalamic area. Ucn expression (green) can be clearly seen within cell body as well as suggested dendritic projection from cell body. Absence of Ucn nuclear staining was confirmed through the use of DAPI (blue). **(m–q)** Snapshot of previous section **(e)** showing; Ucn (green) alone **(m)**, VP (red) alone **(n)**, DAPI (blue) alone **(o)**, Ucn + VP **(p)** and Ucn + VP + DAPI **(q)**. Ucn, urocortin; VP, arginine vasopressin; DAPI, 4′,6-diamidino-2-phenylindole. Scale Bar = 25 μm.

Images taken at higher magnification (Figure 9) revealed some Ucn-like immunoreactivity in VP containing axonal projections. More pronounced colocalisation of Ucn and VP was found in fibres within the ventral SON, suggesting Ucn expression in a subset of dendritic processes. Sequential images were taken through a single vasopressinergic magnocellular neurone to reveal details of intracellular location of Ucn relative to VP (Figure 10). Z-stack analysis revealed that Ucn-like immunoreactivity surrounds the nucleus within the cell body. No colocalisation with DAPI staining was seen, indicating exclusion from the cell nucleus. Robust Ucn expression was found to extend along a process extending outwards from the main cell body. Within the cell body, punctate Ucn staining was found to co-exist to some degree with VP (Figure 10), but to a lesser extent within the projection.

### Ucn Peptide Expression in Adult WKY and SHR HNS

Pixel intensity analysis was used to quantitatively compare Ucn peptide expression in WKY and SHR SON, PVN and posterior pituitary. For both SON (Supplementary Figure 1) and PVN (Supplementary Figure 2), histograms from WKY showed more pixels displaying higher intensity values than those of SHR. Quantitative analysis normalising for differences in total area analysed between images showed a lower average relative intensity of pixels in the SHR SON (45.46 ± 2.07) compared to WKY SON (65.02 ± 3.60) (*p* = 0.0216). Similarly, for the PVN, averaged SHR pixel intensities showed lower relative intensity in the SHR (685.33 ± 27.15) compared to WKY (943.09 ± 55.82) (*p* = 0.042). No differences were seen in the posterior pituitary (Supplementary Figure 3) (SHR, 3,264.54 ± 444.66; WKY, 2,619.16 ± 146.44; *p* = 0.1253).

### Overexpression of Ucn in SHR Does Not Normalise BP

Ucn was over-expressed bilaterally in the SHR PVN by lentiviral mediated gene delivery. Control groups comprised of animals injected with a lentivirus expressing only eGFP. Successful microinjection of lentivirus into PVN was determined through RT-qPCR analysis of expression of both Ucn and eGFP mRNAs (Supplementary Figure 4). Overexpression of Ucn had no effect on endogenous VP or OT mRNA expression (Supplementary Figure 5). Pituitary content of Ucn was also unchanged (Supplementary Figure 6). Overexpression of Ucn had no effect on systolic blood pressure, diastolic blood pressure, heart rate or calculated baroreceptor sensitivity (BRS), as measured by non-invasive radiotelemetry, over the entire duration of the experiment (Supplementary Figure 7). Spectral analysis of blood pressure data revealed a statistically significant decrease (−3.98 mmHg^2^, *p* = 0.041) in total SBP variability between day zero (d0) (7.08 ± 1.75 mmHg^2^) and day 14 (d14) (4.2 ± 0.96 mmHg^2^), with a similar, but not quite significant, trend in VLF SBP (−1.90 mmHg^2^, *p* = 0.063) across these time-points (d0: 4.36 ± 0.65, d14: 2.46 ± 0.65 mmHg^2^) (Supplementary Figure 8). Within the DBP spectra, VLF DBP was significantly decreased (−1.66 mmHg^2^, *p* = 0.036) at d14 (1.86 ± 0.39) compared with d0 (3.52 ± 0.67) (Supplementary Figure 8). No significant differences were found in any HR spectral components and frequencies (Supplementary Figure 9).

## Discussion

We have used Affymetrix microarray analysis to catalogue the transcriptomes of the PVN and SON of normotensive WKY rats and hypertensive SHRs. Comparison of these datasets revealed genes that are differentially up- or down-regulated in the HNS of hypertensive rats compared to normotensive controls. Our data have revealed molecular changes in the HNS between genetically hypertensive and normotensive rats, providing an important catalogue of PVN and SON gene expression that will be a useful resource for researchers interested in study the role of this neuroendocrine system in the pathological phenotypes expressed by SHRs.

Gene Ontology analysis of these datasets revealed significant enrichment of 6 genes involved in the regulation of blood pressure, namely Agtrap, Enpep, Ephx2, Npr3, Pik3r1, and Ucn. Three of these genes (Agtrap, Ephx2, and Ucn) have previously been implicated in the central control of blood pressure (Sellers et al., 2005; Chitravanshi et al., 2013; Yoshida et al., 2014) and were hence validated by RT-qPCR in independent adult samples (Figure 2). Additionally, we also validated these genes in the juvenile SHR and WKY rat brains in order to ask whether differential expression is evident prior to the onset of overt high blood pressure (Figure 2). We found that Agtrap and Ephx2 were both upregulated in the SHR PVN compared to WKY in both juvenile and adult animals. In the SON, Agtrap, and Ephx2 are significantly upregulated in the adult, but not the juvenile SON. In contrast, Ucn is downregulated in juvenile and adult SHR PVN and adult SHR SON. Previous work has shown that Ucn can elicit effects on feeding behaviours (Spina et al., 1996; Moreau et al., 1997) and osmoregulation (Kakiya et al., 1998) when administered centrally. However, although Ucn has been shown to have a role in peripheral cardiovascular control (Takahashi et al., 2004), central actions are not well understood. For that reason, Ucn was selected as the target for further study. Thus, we focussed on the possibility that the reduction in Ucn expression in the SON and PVN of adult SHRs compared to WKY rats might contribute to the high blood pressure seen in that strain.

Immunohistochemical analysis of rat HNS revealed Ucn peptide expression in PVN, SON, ME, and posterior pituitary (Figures 3, 4). Particularly robust expression was found within magnocellular PVN and SON neurons (Figure 3). These results are consistent with previous studies (Bittencourt et al., 1999; Imaki et al., 2001). Similarly, we have shown Ucn expression within both vasopressinergic and oxytocinergic neurons, as also found by Imaki et al. (2001). Measured through the proxy of pixel intensity from confocal images, we found significant reductions in Ucn peptide in the SHR PVN and SON when compared with WKY. Although not of the same magnitude, these results of peptide expression corroborate with our mRNA analyses (Figure 2).

It has been unclear as to whether the Ucn synthesised in the SON and PVN magnocellular cells is transported to the posterior pituitary. Oki et al. (1998) determined by radioimmunoassay that the highest Ucn expression in the CNS was to be found within the pituitary, however specific lobes were not analysed separately. Wong et al. (1996) found Ucn mRNA expression within the neurointermediate and anterior lobes, but not within the posterior lobe. However, Bittencourt et al. (1999) found no cellular expression of peptide across any lobe, but peptide containing fibres in the posterior lobe. Our studies suggest that some Ucn made in the SON and/or PVN may be transported to the posterior pituitary. Within PVN and SON we could not see much axonal Ucn localisation (Figure 9). However, as previously observed (Hara et al., 1997), Ucn-like immunoreactivity was evident in the internal zone of the median eminence. Some of this may originate from hypothalamic magnocellular cells. Further, diffuse Ucn expression was also seen throughout the posterior pituitary, with small areas of punctate staining which may indicate localisation within Herring bodies (Figures 4, 8). Here, some degree of colocalisation of Ucn with VP could be seen and, to a lesser extent, with OT (Figure 8).

Clear expression of Ucn was found within processes in the ventral area of the SON and the lateral PVN (Figure 9). The relative thickness of the projections, anatomical location, and strong peptide immunoreactivity all support dendritic expression. Magnocellular SON neurones are known to extend dendritic projections toward the ventral surface of the brain where they form dense beds (Ludwig and Leng, 2006) containing abundant large dense-core vesicles (Morris and Pow, 1991; Tobin et al., 2012). Many of the Ucn-containing processes demonstrated co-immunofluorescence with VP or OT, both of which, are known to be stored and released from magnocellular dendrites (Bichet, 2014). Indeed, dendrites have been found to be a major source of these and other peptides released into the brain (Ludwig, 1998), and these locally released peptides are involved in pre- and postsynaptic modulation of the electrical activity of magnocellular neurones. It is thus possible that Ucn elicits its central effects through somatodendritic release of Ucn from MCN dendrites acting on cognate receptors (Arima and Aguilera, 2000) to regulate the activity of the same or neighbouring cells.

Chen et al. found that intravenous injection of Ucn into SHR lowered SBP by ∼40 mmHg and functionally and structurally remodelled arteries (Chen et al., 2009). It is tempting to suggest that the HNS might be the source of Ucn that, when released into the blood stream in normotensive animals, reduces blood pressure. When the level of this Ucn is reduced, as in the SHR, blood pressure is increased. We thus tested the hypothesis that lentiviral-mediated restoration of Ucn expression in PVN might lower blood pressure in the SHR. Animals successfully transfected displayed robust and significant increases in expression of appropriate mRNAs pertaining to viral constructs used (Supplementary Figure 4). Ucn overexpression had no effect on PVN VP or OT mRNA levels (Supplementary Figure 5). Ucn peptide levels in the posterior pituitary, as assessed by ELISA, were also unchanged compared to controls (Supplementary Figure 6), suggesting lack of transport from the PVN. Ucn-overexpression produced no significant changes in SBP, DBP, HR, nor BRS (Supplementary Figure 7). We thus conclude that Ucn secreted from the magnocellular HNS has no role in the peripheral control of blood pressure in the adult SHR.

We then analysed spectral components of cardiovascular signals. Impairment of baroreflex function, most notably in regard to BRS, causes significant dysregulation of blood pressure and increases in its variability, and is a well described marker of cardiovascular risk in both human and animal models of essential hypertension (Narkiewicz and Grassi, 2008; Šarenac et al., 2011). Impairment of baroreflex is associated with chronic high blood pressure in the SHR, with the reflex found to quickly reset to a higher functioning pressure following an increase in BP, this resulting in a blunted control over heart rate and sympathetic nerve activity. Significant but transient differences within the Total SBP and VLF DBP spectra of the microinjected PVN Ucn-group between d14 vs. d0 were found. The VLF component, reflecting most of the spectral power in respect to total SBP variability, is known to depend on multiple mechanisms such as efferent sympathetic modulation, myogenic tone, and temperature regulation. In addition, various local or circulating vasoactive factors, such as components of the renin-angiotensin system, nitric oxide, and VP, have been found to modulate the VLF oscillation (Gaudet et al., 1995; Nafz et al., 1996; Japundžić-Žigon, 1998, 2002; Milutinovic et al., 2006). In normotensive rats, VP antagonists enhance VLF BP variability, suggesting that VP contributes to the buffering of VLF BP variability. In the SHR, indications of impairment of this VLF BP buffering effect by VP have been found (Japundžić-Žigon et al., 2004), providing a potential explanation for the reductions in DBP VLF (and also SBP VLF) found at d14 with Ucn overexpression. If Ucn is acting in the PVN in a paracrine or autocrine manner via dendritic release to modulate VP availability or release within the PVN, or via caudal projections to medullary nuclei, it is possible that this would impact the VLF spectra without affecting BP, HR, or BRS. That said, these effects were transitory and provided no beneficial lasting effects on these variables.

In a previous study, we catalogued the transcriptomes of the Sprague Dawley rat SON and PVN, and we documented how they are changed by chronic water deprivation (Hindmarch et al., 2006). Comparison of these datasets with the WHY vs. SHR catalogues presented here revealed no overlap with PVN. However, 7 genes were present in both SON datasets. Of these, 3 are annotated-tropomysin 2 (Tpm2; downregulated 0.48× by dehydration, upregulated 2.31× in SHR vs. WKY), cellular retinol binding-protein type 2 (Crabp2; downregulated 0.35× by dehydration, upregulated 2.06× in SHR vs. WKY) and, interestingly, Ucn (upregulated 2.1× by dehydration, downregulated 0.27× in SHR vs. WKY). It is not known how the Ucn gene responds to dehydration in either WKY or SHR.

Ohno et al. (2018) have shown that the expression of Ucn-like immunoreactivity increases in OT neurones with age, as autonomic dysfunction develops. Paradoxically, we see a decrease in Ucn in PVN and SON of SHRs compared to WKY rats. It would be of interest to find out what happens to the expression of Ucn in these strains as they age.

To conclude, we have found that Ucn is downregulated in juvenile and adult SHR PVN and adult SHR SON. Immunohistochemical analysis revealed that it is mostly co-localised in dendrites of VP and OT containing magnocellular neurones also expressing Ucn receptors, altogether suggesting that UCN may control the same and the neighbouring neurons. Ucn synthetised in the hypothalamus does not play a crucial role in aetiology of hypertension since downregulation of Ucn gene expression had no effect on the mean level of BP. Nevertheless, a transient increase of overall BP variability and VLF BP variability may indicate that Ucn down regulation in SHR may contribution to the aggravation of the disease/may be trigger complications.

## Data Availability Statement

The microarray data has been deposited in the Gene Expression Omnibus (accession: GSE159722).

## Ethics Statement

Experimental procedures were approved by the University of Bristol Ethical Review Committee and performed under the authority of a Project Licence issued by the United Kingdom Home Office in accord with the Animals (Scientific Procedures) Act 1986.

## Author Contributions

CH, DM, JA-R, and JP designed the experiments. AM, CH, DM, MG, ASM, SY, BS, AP, OŠ, BS, and VRA analysed and interpreted the results. CH, ASM, AM, JA-R, VRA, MG, NJ-Ž, OŠ, and SY performed the experiments. CH performed and analysed the transcriptomics. AM, DM, CH, JP, NJ-Ž, and ASM wrote the manuscript with editorial input from all authors.

## Conflict of Interest

The authors declare that the research was conducted in the absence of any commercial or financial relationships that could be construed as a potential conflict of interest.

## Footnotes

1 https://www.nature.com/content/hypertension/index.html

2 http://biostat.mc.vanderbilt.edu/twiki/bin/view/Main/PowerSampleSize

3 www.ncbi.nlm.nih.gov/geo

## References

[B1] Allen A. M. (2002). Inhibition of the hypothalamic paraventricular nucleus in spontaneously hypertensive rats dramatically reduces sympathetic vasomotor tone. *Hypertension* 39 275–280. 10.1161/hy0202.104272 11847197

[B2] Antunes-Rodrigues J. de Castro M. Elias L. L. Valenca M. M. McCann S. M. (2004). Neuroendocrine control of body fluid metabolism. *Physiol. Rev.* 84 169–208. 10.1152/physrev.00017.2003 14715914

[B3] Arima H. Aguilera G. (2000). Vasopressin and oxytocin neurons of hypothalamic supraoptic and paraventricular nuclei co-express mRNA for type-1 and type-2 corticotropin-releasing hormone receptors. *J. Neuroendocrinol.* 12 833–842. 10.1046/j.1365-2826.2000.00528.x 10971808

[B4] Badoer E. (2001). Hypothalamic paraventricular nucleus and cardiovascular regulation. *Clin. Exp. Pharmacol. Physiol.* 28 95–99. 10.1046/j.1440-1681.2001.03413.x 11153547

[B5] Bajić D. Lončar-Turukalo T. Stojičić S. Šarenac O. Bojić T. Murphy D. (2010). Temporal analysis of the spontaneous baroreceptor reflex during mild emotional stress in the rat. *Stress* 13 142–154. 10.3109/10253890903089842 19929315

[B6] Bichet D. G. (2014). Central vasopressin: dendritic and axonal secretion and renal actions. *Clin. Kidney J.* 7 242–247. 10.1093/ckj/sfu050 25852883 PMC4377765

[B7] Bittencourt J. C. Vaughan J. Arias C. Rissman R. A. Vale W. W. Sawchenko P. E. (1999). Urocortin expression in rat brain?: evidence against a pervasive relationship of urocortin-containing projections with targets bearing type 2 CRF receptors. *J. Comp. Neurol.* 312 285–312. 10.1002/(sici)1096-9861(19991220)415:3<285::aid-cne1>3.0.co;2-0 10553117

[B8] Bourque C. W. (1998). Osmoregulation of vasopressin neurons: a synergy of intrinsic and synaptic processes. *Progress Brain Res.* 119 59–76. 10.1016/s0079-6123(08)61562-9 10074781

[B9] Bourque C. W. Oliet S. H. Richard D. (1994). Osmoreceptors, osmoreception, and osmoregulation. *Front. Neuroendocrinol.* 15 231–274. 10.1006/frne.1994.1010 7859914

[B10] Bourque C. W. Voisin D. L. Chakfe Y. (2002). Stretch-inactivated cation channels: cellular targets for modulation of osmosensitivity in supraoptic neurons. *Progress Brain Res.* 139 85–94. 10.1016/s0079-6123(02)39009-5 12436928

[B11] Breyer M. D. Ando Y. (1994). Hormonal signaling and regulation of salt and water transport in the collecting duct. *Annu. Rev. Physiol.* 56 711–739. 10.1146/annurev.ph.56.030194.003431 8010758

[B12] Brownstein M. J. Russell J. T. Gainer H. (1980). Synthesis, transport, and release of posterior pituitary hormones. *Science* 207 373–378. 10.1126/science.6153132 6153132

[B13] Burbach J. P. Luckman S. M. Murphy D. Gainer H. (2001). Gene regulation in the magnocellular hypothalamo-neurohypophysial system. *Physiol. Rev.* 81 1197–1267. 10.1152/physrev.2001.81.3.1197 11427695

[B14] Chen J. Tao J. Zhang R. Xu Y. Soong T. Li S. (2009). Urocortin inhibits mesenteric arterial remodeling in spontaneously hypertensive rats. *Peptides* 30 1117–1123. 10.1016/j.peptides.2009.02.014 19463744

[B15] Chitravanshi V. C. Kawabe K. Sapru H. N. (2013). Mechanisms of cardiovascular actions of urocortins in the hypothalamic arcuate nucleus of the rat. *Am. J. Physiol. Heart Circ. Physiol.* 305 H182–H191. 23686711 10.1152/ajpheart.00138.2013 PMC3726959

[B16] Conrad K. P. Gellai M. North W. G. Valtin H. (1993). Influence of oxytocin on renal hemodynamics and sodium excretion. *Ann. N. Y. Acad. Sci.* 689 346–362. 10.1111/j.1749-6632.1993.tb55559.x 8396871

[B17] Coote J. H. (2005). A role for the paraventricular nucleus of the hypothalamus in the autonomic control of heart and kidney. *Exp. Physiol.* 90 169–173. 10.1113/expphysiol.2004.029041 15604110

[B18] DeVito W. J. Miller M. Sutterer J. R. (1982). Increased secretion of vasopressin and adenosine 3′,5′-monophosphate from hypothalamic-posterior pituitary units of spontaneously hypertensive rats. *Endocrinology* 111 1958–1963. 10.1210/endo-111-6-1958 6291904

[B19] Gaudet E. Blanc J. Elghozi J. L. (1995). Effects of losartan on short-term variability of blood pressure in SHR and WKY rats. *Fundam. Clin. Pharmacol.* 9 30–36. 10.1111/j.1472-8206.1995.tb00262.x 7768485

[B20] Greenwood M. Bordieri L. Greenwood M. P. Rosso Melo M. Colombari D. S. A. Colombari E. (2014). Transcription factor CREB3L1 regulates vasopressin gene expression in the rat hypothalamus. *J. Neurosci.* 34 3810–3820. 10.1523/jneurosci.4343-13.2014 24623760 PMC3951688

[B21] Hara Y. Ueta Y. Isse T. Kabashima N. Shibuya I. Hattori Y. (1997). Increase of urocortin-like immunoreactivity in the rat supraoptic nucleus after dehydration but not food deprivation. *Neurosci. Lett.* 229 65–68. 10.1016/s0304-3940(97)00419-9 9224803

[B22] Hardy S. G. (2001). Hypothalamic projections to cardiovascular centers of the medulla. *Brain Res.* 894 233–240. 10.1016/s0006-8993(01)02053-4 11251196

[B23] Hindmarch C. Yao S. Beighton G. Paton J. Murphy D. (2006). A comprehensive description of the transcriptome of the hypothalamo-neurohypophyseal system in euhydrated and dehydrated rats. *Proc. Natl. Acad. Sci. U.S.A.* 103 1609–1614. 10.1073/pnas.0507450103 16432224 PMC1360533

[B25] Imaki T. Katsumata H. Miyata M. Naruse M. Imaki J. Minami S. (2001). Expression of corticotropin releasing factor (CRF), urocortin and CRF type 1 receptors in hypothalamic-hypophyseal systems under osmotic stimulation. *J. Neuroendocrinol.* 13 328–338. 10.1046/j.1365-2826.2001.00629.x 11264720

[B26] Japundžić-Žigon N. (1998). Physiological mechanisms in regulation of blood pressure fast frequency variations. *Clin. Exp. Hypertens* 20 359–388. 10.3109/10641969809053219 9607401

[B27] Japundžić-Žigon N. (2002). An update on blood pressure short-term variability. *Sci. World J.* 2 320–323. 10.1100/tsw.2002.102 12806019 PMC6009377

[B28] Japundžić-Žigon N. (2013). Vasopressin and oxytocin in control of the cardiovascular system. *Curr. Neuropharmacol.* 11 218–230. 10.2174/1570159x11311020008 23997756 PMC3637675

[B29] Japundžić-Žigon N. Lozić-Đurić M. Šarenac O. Murphy D. (2020). Vasopressin and oxytocin in control of the circulation: an updated review. *Curr. Neuropharmacol.* 18 14–33. 10.2174/1570159x17666190717150501 31544693 PMC7327933

[B30] Japundžić-Žigon N. Milutinović S. Jovanović A. (2004). Effects of nonpeptide and selective V1 and V2 antagonists on blood pressure short-term variability in spontaneously hypertensive rats. *J. Pharmacol. Sci.* 9 47–55. 10.1254/jphs.95.47 15153650

[B31] Kakiya S. Yokoi H. Arima H. Iwasaki Y. Oki Y. Oiso Y. (1998). Central administration of urocortin inhibits vasopressin release in conscious rats. *Neurosci. Lett.* 248 144–146. 10.1016/s0304-3940(98)00357-7 9654364

[B32] Kraly F. S. Coogan L. A. Specht S. M. Trattner M. S. Zayfert C. Cohen A. (1985). Disordered drinking in developing spontaneously hypertensive rats. *Am. J. Physiol.* 248 R464–R470. 3985189 10.1152/ajpregu.1985.248.4.R464

[B33] Latchman D. S. (2002). Urocortin. *Int. J. Biochem. Cell. Biol.* 34 907–910. 12007627 10.1016/s1357-2725(02)00011-0

[B34] Ludwig M. (1998). Dendritic release of vasopressin and oxytocin. *J. Neuroendocrinol.* 10 881–895. 10.1046/j.1365-2826.1998.00279.x 9870745

[B35] Ludwig M. Leng G. (2006). Dendritic peptide release and peptide-dependent behaviours. *Nat. Rev. Neurosci.* 7 126–136. 10.1038/nrn1845 16429122

[B36] Mann S. J. (2018). Neurogenic hypertension: pathophysiology, diagnosis and management. *Clin. Auton. Res.* 28 363–374. 10.1007/s10286-018-0541-z 29974290

[B37] Martin D. S. Haywood J. R. (1998). Reduced GABA inhibition of sympathetic function in renal-wrapped hypertensive rats. *Am. J. Physiol.* 275 R1523–R1529. 9791069 10.1152/ajpregu.1998.275.5.R1523

[B38] McKinley M. J. Mathai M. L. McAllen R. M. McClear R. C. Miselis R. R. Pennington G. L. (2004). Vasopressin secretion: osmotic and hormonal regulation by the lamina terminalis. *J. Neuroendocrinol.* 16 340–347. 10.1111/j.0953-8194.2004.01184.x 15089972

[B39] Mecawi A. S. Vilhena-Franco T. Fonseca F. V. Reis L. C. Elias L. L. Antunes-Rodrigues J. (2013). The role of angiotensin II on sodium appetite after a low-sodium diet. *J. Neuroendocrinol.* 25 281–291. 10.1111/j.1365-2826.2012.02388.x 23002791

[B40] Mecawi Ade S. Ruginsk S. G. Elias L. L. Varanda W. A. Antunes-Rodrigues J. (2015). Neuroendocrine regulation of hydromineral homeostasis. *J. Compr. Physiol.* 5 1465–1516. 10.1002/cphy.c140031 26140725

[B41] Milutinović S. Murphy D. Japundžić-Žigon N. (2006). The role of central vasopressin receptors in the modulation of autonomic cardiovascular controls: a spectral analysis study. *Am. J. Physiol. Regulatory* 291 R1579–R1591. 10.1152/ajpregu.00764.2005 17085750

[B42] Moreau J. L. Kilpatrick G. Jenck F. (1997). Urocortin, a novel neuropeptide with anxiogenic-like properties. *Neuroreport* 8 1697–1701. 10.1097/00001756-199705060-00027 9189917

[B43] Morishita R. Higaki J. Nakamura Y. Aoki M. Yamada K. Moriguchi A. (1995). Effect of an antihypertensive drug on brain angiotensin II levels in renal and spontaneously hypertensive rats. *Clin. Exp. Pharmacol. Physiol.* 22 665–669. 10.1111/j.1440-1681.1995.tb02085.x 8542682

[B44] Morris J. F. Pow D. V. (1991). Widespread release of peptides in the central nervous system: quantitation of tannic acid-captured exocytoses. *Anat. Rec.* 231 437–445. 10.1002/ar.1092310406 1793174

[B45] Morris M. Wren J. A. Sundberg D. K. (1981). Central neural peptides and catecholamines in spontaneous and DOCA/salt hypertension. *Peptides* 2 207–211. 10.1016/s0196-9781(81)80035-6 6117062

[B46] Nafz B. Just A. Stauss H. M. Wagner C. D. Ehmke H. Kirchheim H. R. (1996). Blood-pressure variability is buffered by nitric oxide. *J. Auton. Nerv. Syst.* 57 181–183. 10.1016/0165-1838(95)00080-1 8964946

[B47] Narkiewicz K. Grassi G. (2008). Impaired baroreflex sensitivity as a potential marker of cardiovascular risk in hypertension. *J. Hypertension* 26 1303–1304. 10.1097/hjh.0b013e328305e1a5 18551001

[B48] Ohno S. Hashimoto H. Fujihara H. Fujiki N. Yoshimura M. Maruyama T. (2018). Increased oxytocin-monomeric red fluorescent protein 1 fluorescent intensity with urocortin-like immunoreactivity in the hypothalamo-neurohypophysial system of aged transgenic rats. *Neurosci. Res.* 128 40–49. 10.1016/j.neures.2017.08.001 28859972

[B49] Oki Y. Iwabuchi M. Masuzawa M. Watanabe F. Ozawa M. Iino K. (1998). Distribution and concentration of urocortin, and effect of adrenalectomy on its content in rat hypothalamus. *Life Sci.* 62 807–812. 10.1016/s0024-3205(97)01182-x 9496698

[B50] Panyasrivanit M. Greenwood M. P. Murphy D. Isidoro C. Prasert Auewarakul P. Smith D. R. (2011). Induced autophagy reduces virus output in dengue infected monocytic cells. *Virology* 418 74–84. 10.1016/j.virol.2011.07.010 21813150

[B51] Paxinos G. Watson C. (2013). *The Rat Brain in Stereotaxic Coordinates*, 7th Edn. Amsterdam: Elsevier.

[B52] Pérez-Delgado M. M. Carmona-Calero E. Marrero-Gordillo N. Pérez-González H. Castañeyra-Perdomo A. (2000). Effect of hypertension on the angiotensin II fibres arriving at the posterior lobe of the hypophysis of the rat. An immunohistochemical study. *Histol. Histopathol.* 15 73–77. 10668197 10.14670/HH-15.73

[B53] Pfaffl M. W. (2001). A new mathematical model for relative quantification in real-time RT–PCR. *Nucleic Acids Res.* 29:e45. 10.1093/nar/29.9.e45 PMC55695 11328886

[B54] Qin C. Li J. Tang K. (2018). The paraventricular nucleus of the hypothalamus: development. function, and human diseases. *Endocrinology* 159 3458–3472. 10.1210/en.2018-00453 30052854

[B55] Šarenac O. Lozić M. Drakulić S. Bajić D. Paton J. F. Murphy D. (2011). Autonomic mechanisms underpinning the stress response in borderline hypertensive rats. *Exp. Physiol.* 96 574–589. 10.1113/expphysiol.2010.055970 21421701 PMC3272224

[B56] Sawchenko P. E. Swanson L. W. (1982). Immunohistochemical identification of neurons in the paraventricular nucleus of the hypothalamus that project to the medulla or to the spinal cord in the rat. *J. Comparative Neurol.* 205 260–272. 10.1002/cne.902050306 6122696

[B57] Saxena T. Ali A. O. Saxena M. (2018). Pathophysiology of essential hypertension: an update. *Exp. Rev. Cardiovasc Ther.* 16 879–887. 10.1080/14779072.2018.1540301 30354851

[B58] Sellers K. W. Sun C. Diez-Freire C. Waki H. Morisseau C. Falck J. R. (2005). Novel mechanism of brain soluble epoxide hydrolase-mediated blood pressure regulation in the spontaneously hypertensive rat. *FASEB J.* 19 626–628. 15659536 10.1096/fj.04-3128fje

[B59] Simms A. E. Paton J. F. R. Pickering A. E. Allen A. (2009). Amplified respiratory-sympathetic coupling in neonatal and juvenile spontaneously hypertensive rats: does it contribute to hypertension? *J. Physiol.* 587 597–610. 10.1113/jphysiol.2008.165902 19064613 PMC2670083

[B60] Skelton K. H. Owens M. J. Nemeroff C. B. (2000). The neurobiology of urocortin. *Regul. Pept.* 93 85–92. 10.1016/s0167-0115(00)00180-4 11033056

[B61] Sladek C. D. Devine M. A. Felten S. Y. Aravich P. F. Blair M. L. (1988). Abnormalities in hypothalamic and neurohypophysial vasopressin content are not a consequence of hypertension in the spontaneously hypertensive rat. *Brain Res.* 445 39–46. 10.1016/0006-8993(88)91071-2 3130152

[B62] Spina M. Merlo-Pich E. Chan R. K. W. Basso A. M. (1996). Appetite-suppressing effects of urocortin, a CRF-related neuropeptide. *Science* 273 1561–1564. 10.1126/science.273.5281.1561 8703220

[B63] Stengel A. Taché Y. (2014). CRF and urocortin peptides as modulators of energy balance and feeding behavior during stress. *Front. Neurosci.* 8:52. 10.3389/fnins.2014.00052 24672423 PMC3957495

[B64] Stojicić S. Milutinović-Smiljanić S. Sarenac O. Milosavljević S. Paton J. F. R. Murphy D. (2008). Blockade of central vasopressin receptors reduces the cardiovascular response to acute stress in freely moving rats. *Neuropharmacology* 54 824–836. 10.1016/j.neuropharm.2007.12.013 18339407

[B65] Takahashi K. Totsune K. Murakami O. Shibahara S. (2004). Urocortins as cardiovascular peptides. *Peptides* 25 1723–1731. 10.1016/j.peptides.2004.04.018 15476939

[B66] Tobin V. Schwab Y. Lelos N. Onaka T. Pittman Q. J. Ludwig M. (2012). Expression of exocytosis proteins in rat supraoptic nucleus neurones. *J. Neuroendocrinol.* 24 629–641. 10.1111/j.1365-2826.2011.02237.x 21988098 PMC3569506

[B67] Trippodo N. C. Frohlich E. D. (1991). Similarities of genetic (spontaneous) hypertension. Man and rat. *Circ. Res.* 48 309–319. 10.1161/01.res.48.3.309 7460205

[B68] Turuk Turukalo T. L. Bajić D. Japundžić-Žigon N. (2011). Temporal sequence parameters in isodistributional surrogate data: model and exact expressions. *IEEE Trans. Bio-Med. Eng.* 58 16–24. 10.1109/tbme.2010.2083661 20923727

[B69] van Tol H. H. van den Buuse M. de Jong W. Burbach J. P. (1988). Vasopressin and oxytocin gene expression in the supraoptic and paraventricular nucleus of the spontaneously hypertensive rat (SHR) during development of hypertension. *Brain Res.* 464 303–311. 10.1016/0169-328x(88)90039-3 3233490

[B70] Wong M. L. Al-Shekhlee A. Bongiorno P. B. Esposito A. Khatri P. Sternberg E. M. (1996). Localization of urocortin messenger RNA in rat brain and pituitary. *Mol. Psychiatry* 1 307–312. 9118356

[B71] World Health Organization [WHO] (2019). Available online at: https://www.who.int/news-room/fact-sheets/detail/hypertension (accessed September 13, 2019).

[B72] World Heart Federation [WHF] (2017). Available online at: https://www.world-heart-federation.org/resources/key-facts/ (accessed May 30, 2017).

[B73] Yang Z. Coote J. H. (1999). Influence of the hypothalamus paraventricular nucleus on cardiovascular neurones in the rostral ventrolateral medulla of the rat. *J. Physiol.* 513(Pt 2) 521–530. 10.1111/j.1469-7793.1998.521bb.x 9807000 PMC2231294

[B74] Yoshida M. Watanabe Y. Yamanishi K. Yamashita A. Yamamoto H. Okuzaki D. (2014). Analysis of genes causing hypertension and stroke in spontaneously hypertensive rats: gene expression profiles in the brain. *Int. J. Mol. Med.* 33 887–896. 10.3892/ijmm.2014.1631 24452243

[B75] Zhang Z. Bourque C. W. (2003). Osmometry in osmosensory neurons. *Nat. Neurosci.* 6 1021–1022. 10.1038/nn1124 12973356

[B76] Zhou J. J. Ma H. J. Shao J. Y. Pan H. L. Li D. P. (2019). Impaired hypothalamic regulation of sympathetic outflow in primary hypertension. *Neurosci. Bull.* 35 124–132. 10.1007/s12264-018-0316-5 30506315 PMC6357282

