# Supplementary material for: Transcriptome Analysis Reveals Downregulation of Urocortin Expression in the Hypothalamo-Neurohypophysial System of Spontaneously Hypertensive Rats
Source: Front Physiol. 2021 Mar 17;11:599507. doi: 10.3389/fphys.2020.599507 (PMC8011454; doi:10.3389/fphys.2020.599507)
Supplement: Supplementary file 1 [file Table_1.docx]

**Supplementary Table 1.**

| **mRNA** | **Fwd/Rev** | **Primer Sequence (5' - 3')** |
| --- | --- | --- |
| *Agtrap* | Fwd | CAGCTTGGCCCTTGTTCTCCAG |
|  | Rev | CATCCTCAGCTTGCTGCTGAAG |
| *Ucn* | Fwd | TCCAGGATCCGAATCTGCGAT |
|  | Rev | AAGGGTCAAGGCTTTCTGGAC |
| *Ephx2* | Fwd | GAGACTTCCTACTTGGCGCT |
|  | Rev | AGTGGTACCCACTGGGAAAATG |
| *VP* | Fwd | TGCCTGCTACTTCCAGAACTGC |
|  | Rev | AGGGGAGACACTGTCTCAGCTC |
| *OT* | Fwd | TGAGCAGGAGGGGGCCTAGC |
|  | Rev | TGCAAGAGAAATGGGTCAGTGGC |
| *Rpl19* | Fwd | GCGTCTGCAGCCATGAGTA |
|  | Rev | TGGCATTGGCGATTTCGTTG |

Primer pairs used for qPCR analysis. Fwd; Forward, Rev; Reverse, Agtrap; Type-I Angiotensin II receptor-associated protein, Ucn; Urocortin, NmB; Neuromedin B, Ephx2; Epoxide Hydrolase 2, VP; Arginine Vasopressin, OT; Oxytocin, Rpl19; Ribosomal Protein L19.

**Supplementary Table 2.**

| **Primary** | **Supplier** | **Cat. No.** | **Host** | **Dilution** |
| --- | --- | --- | --- | --- |
| Urocortin (R-20) | Santa Cruz | sc-1825 | Goat | 1-100 |
| Vasopressin (neurophysin II; PS41) | Harold Gainer | n/a | Mouse | 1-250 |
| Oxytocin (neurophysin I; PS38) | Harold Gainer | n/a | Mouse | 1-250 |
| Glial Fibrillary Acidic Protein | Santa Cruz | sc-65343 | Mouse | 1-250 |
| **Secondary** |  |  |  |  |
| Biotin Conjugated anti-Goat IgG (H+L) | Vector Labs | BA-5000 | Horse | 1-500 |
| Streptvidin Alexa Fluor 488 Conjugate | ThermoFisher | S11223 | n/a | 1-500 |
| Streptvidin Alexa Fluor 594 Conjugate | ThermoFisher | S11227 | n/a | 1-500 |
| anti-Goat IgG (H+L) Alexa Fluor 594 | ThermoFisher | A11080 | Rabbit | 1-500 |
| anti-Mouse IgG (H+L) Alexa Fluor 488 | ThermoFisher | A-21202 | Donkey | 1-500 |
| anti-Mouse IgG (H+L) Alexa Fluor 594 | ThermoFisher | A-21203 | Donkey | 1-500 |

Primary and secondary antibodies used for immunofluorescence.

**Supplementary Table 3.**

***Microarray data.***

See Supplementary Table 3.xls file

GeneSpring® was used to compile a list of genes called present (P) in all five independent WKY PVN (3A), SHR PVN (3B), WKY SON (3C) and SHR SON (3D) samples. All marginal, absent or unknown calls were excluded. The corresponding gene lists are transcriptome catalogues that, with a high degree of confidence, represent comprehensive descriptions of the RNA populations expressed in various cardiovascular control brain regions in either WKY rats or SHRs. The following information is provided:

• Affymetrix probe set identifier (Affy ID)

• Average raw signal

• Gene symbol

• Genbank ID

• Gene description

GeneSpring® was used to combine the WKY and SHR gene lists to produce gene catalogues that, for each tissue, represent transcripts called present in all five experiments of either the WKY or the SHR strain (WKY+SHR-P). These combined lists were then used as the basis for further filtering and statistical analysis. Firstly, for each tissue, lists were filtered to identify genes that are putatively increased or decreased by at least 2-fold in the SHR compared to WKY. These lists were then used to statistically assess SHR vs WKY changes (Welsh t-test, p<0.05, with Benjamini-Hochberg [BH] multiple test correction). The following information is provided:

- Affymetrix probe set identifier (Affy ID)
- Fold difference
- Gene symbol
- Genbank identifier
- Gene description

**Supplementary Table 4.**

***SON GO analysis data***

See Supplementary Table 4.xlsx file

The STRING database (string-db.org) was used to ask if the differentially expressed transcripts identified in the SON could be classified according to enriched biological process gene ontology (GO) terms. The following information is provided:

- GO term ID
- GO term description
- Observed gene count
- Background gene count
- False discovery rate
- Gene symbols


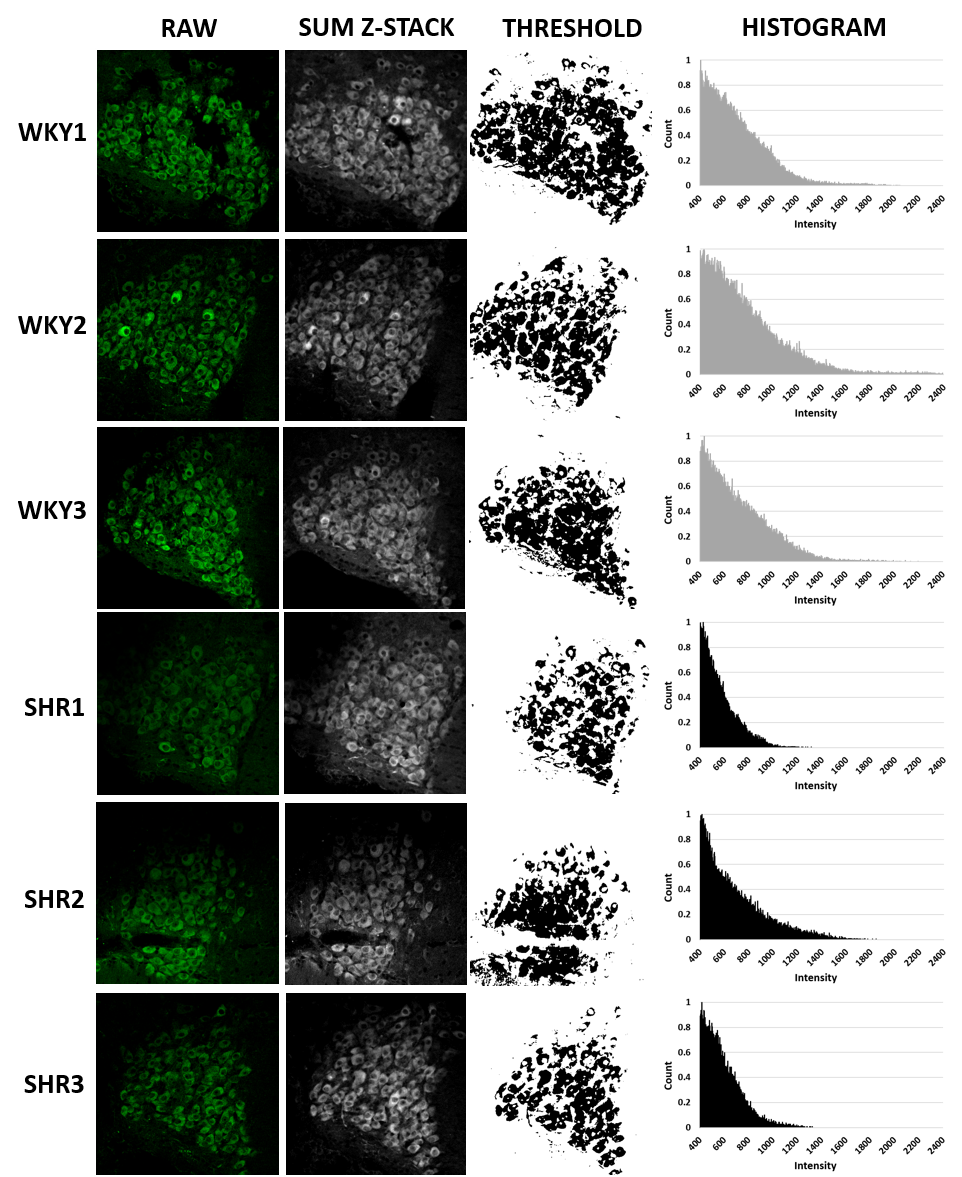


Supplementary Figure 1. Analysis of Urocortin signal intensity in SON. Analysis of Ucn signal intensity from WKY (n=3) and SHR (n=3) coronal slice samples from SON of the HNS. Number of slices per z-stack = 10.


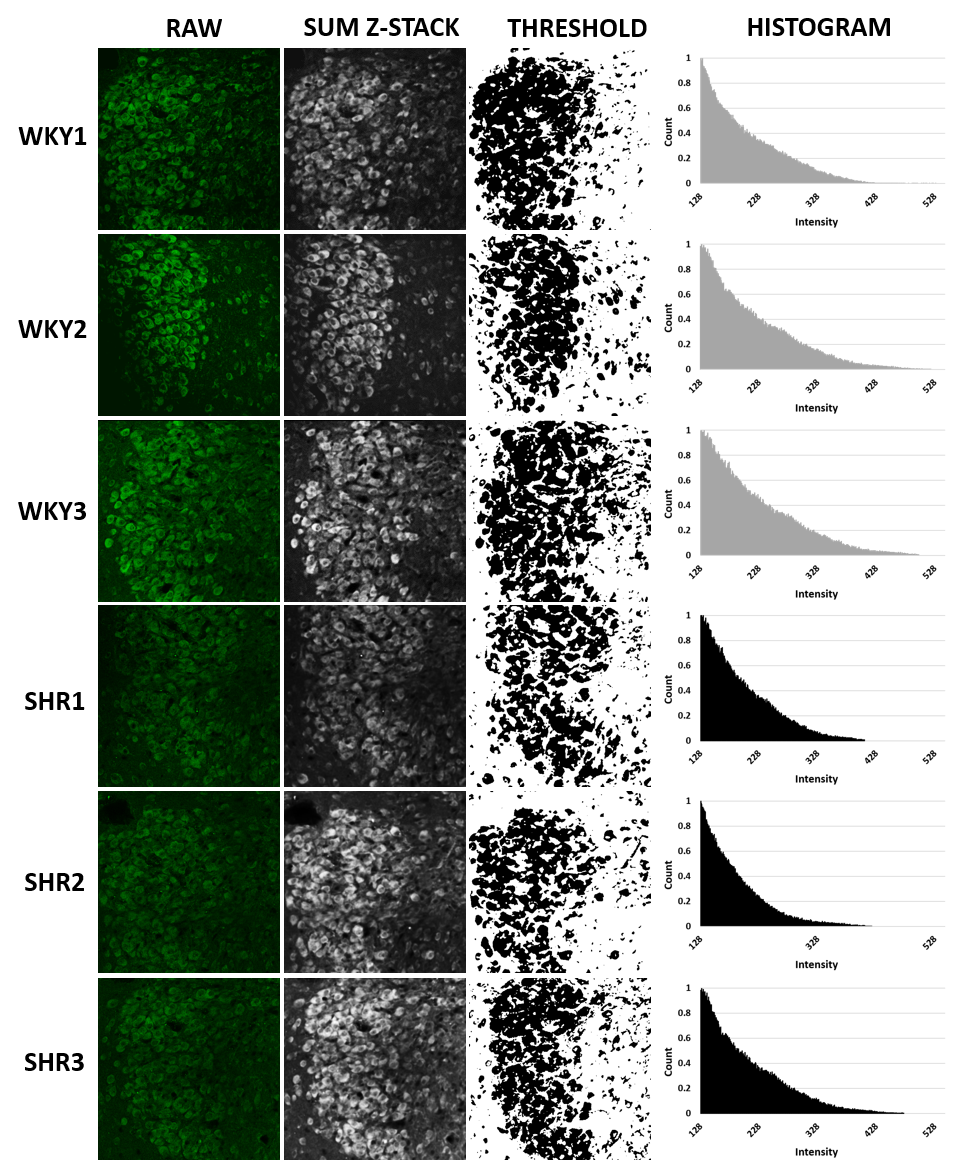


Supplementary Figure 2. Analysis of Urocortin signal intensity in PVN. Analysis of Ucn signal intensity from WKY (n=3) and SHR (n=3) coronal slice samples from PVN of the HNS. Number of slices per z-stack = 5.


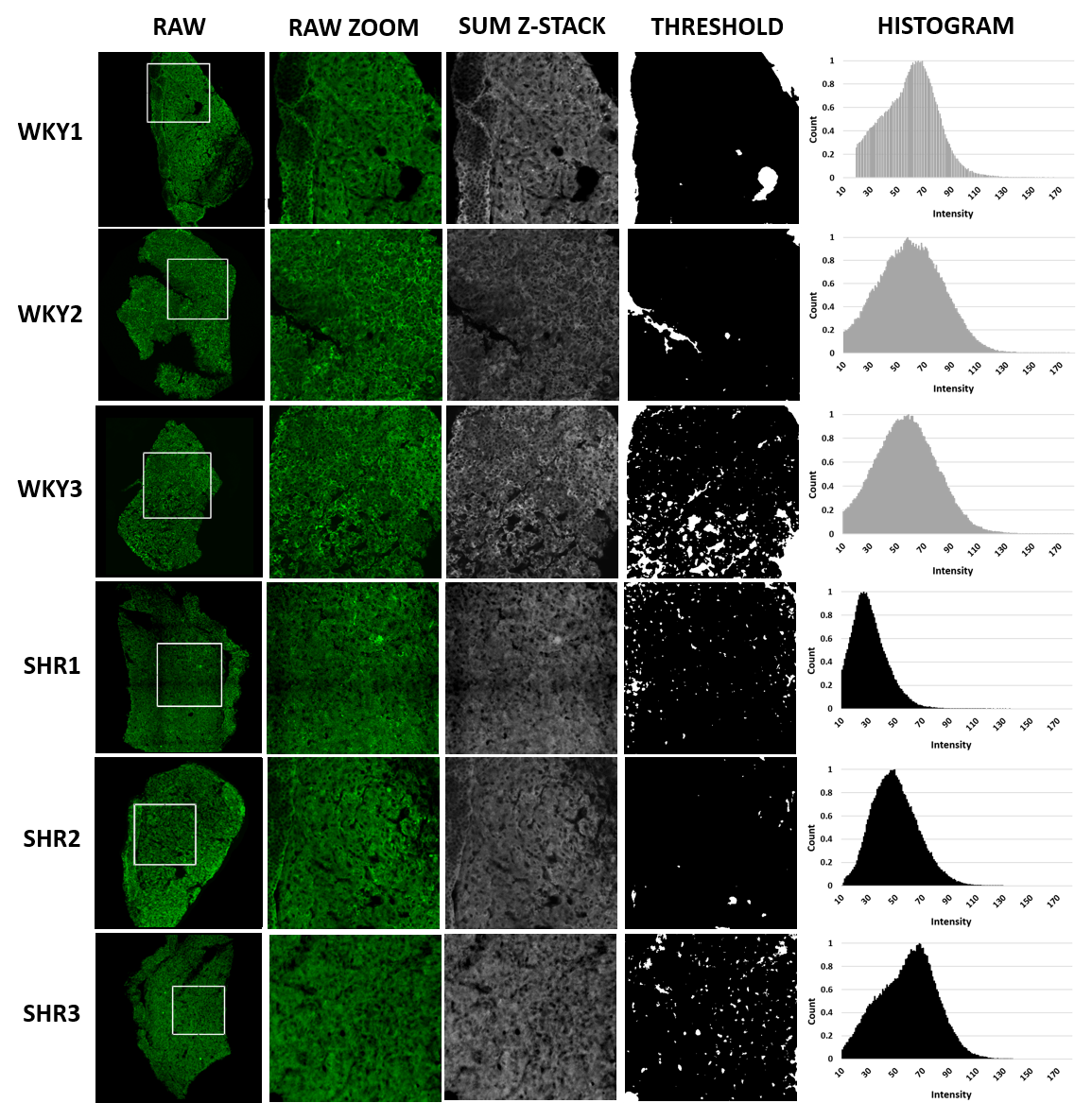


Supplementary Figure 3. Analysis of Urocortin signal intensity in posterior pituitary. Analysis of Ucn signal intensity from WKY (n=3) and SHR (n=3) slice samples from the posterior pituitary of the HNS. Number of slices per z-stack = 1.

Supplementary Figure 4. Relative expression levels of transgene mRNAs in adult SHR PVN following lentiviral microinjection. Ucn mRNA expression was increased significantly in the PVN of the Ucn-transfected group compared to the EGFP group. Likewise, EGFP mRNA expression was increased significantly in the PVN of the EGFP-transfected group compared to the Ucn group. Ucn; Urocortin, EGFP; Enhanced green fluorescent protein. Asterisk’s denote significance as follows; * = p ≤ 0.05, ** = p ≤ 0.01. Error bars = Standard Error of Mean (S.E.M).

Supplementary Figure 5. Relative expression levels of VP and OT RNAs following lentiviral overexpression of either Ucn or EGFP in PVN of SHRs. No significant difference in expression of either VP or OT was found between Ucn or EGFP groups in the PVN of transfected SHRs. VP; Arginine Vasopressin, OT; Oxytocin. Error bars = Standard Error of Mean (S.E.M.).

Supplementary Figure 6. Ucn Concentration in the pituitary following bilateral PVN injection of lentivirus. Viruses overexpressing either Ucn or EGFP. An ELISA performed on pituitary samples from either Ucn or EGFP transduced SHR groups found no significant difference in concentration of Ucn. Ucn; Urocortin, eGFP; Enhanced green fluorescent protein. Error bars = Standard Error of Mean (S.E.M.).

Supplementary Figure 7. Cardiovascular parameters of SHR rats following lentiviral microinjection into PVN. EGFP/Ucn -overexpressing lentivirus. Overexpression of Ucn in the PVN of SHRs showed no clear or statistically significant effects over any cardiovascular parameter measured (SBP, DBP, HR) or calculated (BRS). SBP, systolic BP; DBP, diastolic BP; HR, heart rate; BRS, baroreflex sensitivity. Values are mean of EGFP group (n=4) and Ucn group (n=5) ± S.E.M.

Supplementary Figure 8. Components of BP short-term variability in SHR rats following lentiviral microinjection into PVN. Either EGFP/Ucn -overexpressing lentivirus. Overexpression of Ucn in the PVN of SHRs elicited a statistically significant decrease in Total SBP measured at d14 compared to at transfection (d0), however this effect was not found at later time-points. No other significant effects were found across systolic frequency range of LF or HF, however a strong trend decrease was seen in the VLF spectrum. Within the diastolic frequency range a reduction in Total DBP was observed at d14 vs d0 in the SHR, but this did not reach significance. Within the VLF spectra however, a significant decrease was found in the Ucn group at d14 vs d0, however this effect was not observed at later time-points. No other significant differences were observed across time-points or between groups in LF DBP or HF DBP ranges. Total SBP, total SBP variability; Total DBP, total DBP variability; VLF SBP, very low-frequency SBP; VLF DBP, very low-frequency DBP variability; LF SBP, low-frequency SBP variability; LF DBP, low-frequency DBP variability; HF SBP, high-frequency SBP variability, HF DBP, low-frequency DBP variability. Values are Mean of EGFP group rats (n=4) and Ucn group rats (n=5) ± S.E.M. * = p < 0.05 vs transfection (d0).

Supplementary Figure 9. Components of HR short-term variability in SHR rats; following microinjection of EGFP/Ucn -overexpressing lentivirus into PVN. Within the frequency ranges of HR, no significant differences were observed between d0 and later time-points in either the Ucn or EGFP groups. Ucn group showed an overall lower average across all HR frequency spectra at all time-points measured compared to EGFP, this reduction was never found to reach significance however. The LF/HF_HR_ ratio showed no clear pattern or significant differences between groups or time-points. Total HR, total HR variability; VLF HR, very low-frequency HR; LF HR, low-frequency HR variability; HF HR, high-frequency HR variability, LF/HF_HR_, low-frequency HR/high-frequency HR. Values are mean of EGFP group (n=4) and Ucn group (n=5) ± S.E.M.
